# Inhibition of fat cell differentiation in 3T3-L1 pre-adipocytes by all-trans retinoic acid: Integrative analysis of transcriptomic and phenotypic data

**DOI:** 10.1016/j.bdq.2016.11.001

**Published:** 2016-11-21

**Authors:** Katharina Stoecker, Steffen Sass, Fabian J. Theis, Hans Hauner, Michael W. Pfaffl

**Affiliations:** a Animal Physiology and Immunology, School of Life Sciences Weihenstephan, Technical University of Munich, Freising, Germany; b Institute of Computational Biology, Helmholtz Center Munich, German Research Center for Environmental Health, Oberschleißheim, Germany; c Department of Mathematics, Technical University of Munich, Garching, Germany; d Else-Kröner-Fresenius-Centre for Nutritional Medicine, ZIEL Research Center for Nutrition and Food Sciences, Technical University of Munich, Freising, Germany; e Else Kröner-Fresenius-Centre for Nutritional Medicine, Klinikum rechts der Isar, Technical University of Munich, Munich, Germany

**Keywords:** All-trans retinoic acid (ATRA), Adipogenesis, 3T3-L1 cells, MicroRNA–mRNA interaction, Regulation of biological pathways, Multiple linear regression models

## Abstract

The process of adipogenesis is controlled in a highly orchestrated manner, including transcriptional and post-transcriptional events. In developing 3T3-L1 pre-adipocytes, this program can be interrupted by all-trans retinoic acid (ATRA). To examine this inhibiting impact by ATRA, we generated large-scale transcriptomic data on the microRNA and mRNA level. Non-coding RNAs such as microRNAs represent a field in RNA turnover, which is very important for understanding the regulation of mRNA gene expression. High throughput mRNA and microRNA expression profiling was performed using mRNA hybridisation microarray technology and multiplexed expression assay for microRNA quantification. After quantitative measurements we merged expression data sets, integrated the results and analysed the molecular regulation of *in vitro* adipogenesis. For this purpose, we applied local enrichment analysis on the integrative microRNA-mRNA network determined by a linear regression approach. This approach includes the target predictions of TargetScan Mouse 5.2 and 23 pre-selected, significantly regulated microRNAs as well as Affymetrix microarray mRNA data. We found that the cellular lipid metabolism is negatively affected by ATRA. Furthermore, we were able to show that microRNA 27a and/or microRNA 96 are important regulators of gap junction signalling, the rearrangement of the actin cytoskeleton as well as the citric acid cycle, which represent the most affected pathways with regard to inhibitory effects of ATRA in 3T3-L1 preadipocytes. In conclusion, the experimental workflow and the integrative microRNA–mRNA data analysis shown in this study represent a possibility for illustrating interactions in highly orchestrated biological processes. Further the applied global microRNA–mRNA interaction network may also be used for the pre-selection of potential new biomarkers with regard to obesity or for the identification of new pharmaceutical targets.

## 1 Introduction

Dietary behaviour has a strong influence on body energy metabolism. White adipose tissue (WAT) is the major storage depot for excess energy and is involved in energy homeostasis. WAT is mainly composed of mature adipocytes and pre-adipocytes as well as other cell types. An expansion of adipose tissue is usually because of an increase in fat cell volume, but in the case of severe forms of obesity, it is also characterised by fat cell hyperplasia caused by preadipocyte differentiation. This process is regulated in a highly complex manner and includes many regulatory components [1]. However, the enlargement of body fat mass and an increase in body mass index (BMI) depends on a chronic positive energy balance [2].

Adipogenesis, the process of fat cell differentiation can be affected by various endogenous and/or exogenous factors. Accordingly, nutrients or other compounds affecting adipogenesis exert different modes of action by influencing factors such as adipocyte size and number, and the secretion of hormones regulating hunger, satiety or energy expenditure [3]. Fructose, nicotine, pesticides, industrial chemicals or pharmaceuticals like estradiol are able to mediate pro-adipogenic functions in mammals and are called obesogenic compounds [3], [4]. On the other hand, anti-adipogenic compounds also exist in nature. All-trans retinoic acid (ATRA), a vitamin A derivate, is capable of modulating differentiation [5], [6], [7]. Thereby, the differentiation of osteoblasts [8] and myoblasts [9] is induced, whereas the development of adipocytes is inhibited [10], [11], [12]. The effects of ATRA strongly depend on its concentration, duration of action and target cell type [13]. Retinol and carotenoids, the precursors of retinoic acid, are found in many foods, e.g. meat, milk and vegetables, and in high concentrations in carrots. After absorption from the diet, retinoic acid is transported to various tissues including, WAT, and is metabolised inside the cells [5], [7]. The effects of ATRA on adipogenesis have be examined in cell culture models e.g. the mouse 3T3-L1 pre-adipocyte cell line [14]. In confluent growth-arrested 3T3-L1 pre-adipocytes, an adipogenic stimulus initiates a limited clonal expansion before the cells enter the phase of terminal differentiation, including lipid accumulation [15], [16], [17]. For this, the sequential induction of the transcription factors *Cebpα*, *Cebpβ* [18] and *Pparγ* [19], [20], [21] is necessary. Thereafter, adipocyte specific genes and enzymes are induced [22], and cell morphology is changed by reorganisation of the cytoskeleton from a fibroblast-like appearance towards a spherical shape [22], [23]. Recent data suggest that the development of mature adipocytes is initiated by transcriptional as well as post-transcriptional changes conveyed by small non-coding RNAs. MicroRNAs (miRs) are small non-coding RNA species, and regulation takes place through both, mRNA degradation or suppression of translation [24], [25]. This mechanism was initially discovered in plants and *Caenorhabditis elegans* by Lee et al. and was reported to be associated with the regulation of gene expression and cell differentiation and with guarding organisms against external nucleotide sequences such as viruses, transposons or parasites [26].

The present study aimed to further study the molecular mechanisms induced by ATRA in 3T3-L1 mouse preadipocytes at the transcriptome and phenotypic levels in a high-resolution time frame. Therefore, we used the method of oil-red-O staining for phenotyping and microarray technology, reverse-transcription quantitative polymerase chain reaction (RT-qPCR) and a multiplexed assay for expression profiling at both the mRNA and miR levels. Pre-adipocytes were treated with ATRA in a time frame from 0 h to 288 h (12 days) post-treatment. In addition, to generate a comprehensive picture of regulatory networks of the physiological processes, we combined these high-throughput transcriptional data sets to create a two-level regulatory mRNA-miR network of transcriptomic data.

## 2 Materials and methods

### 2.1 Cell culture for maintenance

For all experiments, the mouse preadipocyte cell line 3T3-L1 (ATCC^®^/LGC Standards GmbH, Wesel, Germany) was used, and cells were cultured as described by the supplier. The cells were maintained in T175 flasks (Nalgene Nunc International/Fisher Scientific, Schwerte, Germany) with fibroblast medium consisting of 500 ml Dulbecco’s modified Eaglés medium (DMEM) (LGC Standards GmbH, Wesel, Germany), 10% new born calf serum (PAN Biotech GmbH, Aidenbach, Germany) and 1% penicillin-streptomycin (Invitrogen™, Darmstadt, Germany). Cells were subcultured every 3 days. Following this, the cells were washed with phosphate-buffered saline (PBS) (PAA Laboratories GmbH, Cölbe, Germany), and the cell layer was detached with 0.25% Trypsin–EDTA (Life Technologies GmbH, Darmstadt, Germany). Cell culture was performed at 37 °C in a humidified 5% CO_2_ incubator.

### 2.2 Experimental cell culture

Initially, cells were resuspended in fibroblast medium and 1.8 × 10^5^ cells were seeded per six wells (Nalgene Nunc International/Fisher Scientific, Schwerte, Germany). After 5 days, when pre-confluence was reached, the fibroblast medium was renewed and the cells were cultured for another 2 days. The fibroblast medium was replaced by differentiation medium (DMI), containing 10% foetal bovine serum (FBS) (PAN Biotech GmbH, Aidenbach, Germany), 1% penicillin–streptomycin, 0.5 mM 3-Isobutyl-1-methylxanthin (IBMX) (Sigma Aldrich, Taufkirchen, Germany), 1 μM dexamethasone (DEX) (Sigma Aldrich) and 1 μg/ml insulin (Sigma Aldrich) in 500 ml DMEM. The 3T3-L1 preadipocytes were cultivated with DMI in the presence and absence of 3 μM ATRA (Sigma Aldrich, Taufkirchen, Germany). After 96 h, DMI was replaced by growth medium (±3 μM ATRA) containing 10% FBS, 1% penicillin–streptomycin and 1 μg/ml insulin in 500 ml DMEM. The medium was renewed every 2 days. ATRA-treated and −untreated 3T3-L1 cells were harvested after 0, 2, 4, 7, 10, 24, 48 and 96 h and again after 288 h (12 days).

### 2.3 Evaluation of RNA quality

Cellular RNA of ATRA-treated and untreated 3T3-L1 preadipocytes was extracted using the RNeasy Kit (Qiagen, Hilden, Germany), as described by the supplier. RNA was eluted in RNase-free water. The RNA concentration and purity were determined using the Spectrophotometer NanoDrop1000 (NanoDrop products, Wilmington, USA) and Bioanalyzer 2100 (Agilent Technologies, Mannheim, Germany).

### 2.4 cDNA synthesis

For gene expression profiling, 500 ng of total RNA per sample was reverse transcribed into first-strand cDNA. For cDNA synthesis, the Moloney murine leukaemia virus reverse transcriptase H- (M-MLV RT H-) (Promega, Mannheim, Germany), 10 mM dNTPs and 50 μM hexamer primers were utilised in a total volume of 31 μl. The reaction mix was incubated for 20 min at 21 °C, following which the cDNA synthesis step was performed for 120 min at 48 °C. The reaction was stopped by 2-min incubation at 90 °C.

For the reverse transcription of mature miRs, we used the miScript RT Kit (Qiagen), as described by the supplier. The total volume of each reaction was 20 μl. We used 300 ng total RNA. The RT reactions were performed by incubation for 1 h at 37 °C, and the cDNA reaction was stopped by incubation at 95 °C for 5 min. Thereafter, cDNA was diluted 1:4 with RNase-free water and stored at −20 °C.

### 2.5 RT-qPCR for mRNA quantification

RT-qPCR was performed using a CFX384 Touch real-time detection cycler (Bio-Rad Laboratories, Munich, Germany), and the SsoFast EvaGreen Supermix (Bio-Rad Laboratories) was used for gene expression profiling. Gene primers (200 nM) and 1 μl of first-strand cDNA were added to the master mix. From the entire set of all quantified genes the most stable mRNAs were selected for data normalisation. We applied the geometrical average of the following four stably expressed reference genes: Glyceraldehyde-3-phosphate dehydrogenase (*Gapdh*); Non-POU-domain-containing Octamer-binding protein (*Nono*); Beta Actin (*Actb*) [27]*;* and Importin 8 (*Ipo8*). We quantified the expression of following genes of interest: Peroxisome proliferator-activated receptor gamma (*Pparγ*); CCAAT/enhancer-binding protein (C/EBP), alpha (*Cebpα*); CCAAT/enhancer-binding protein (C/EBP), beta (*Cebpβ*); sterol regulatory element binding transcription factor 1 (*Srebf1*) and retinoid X receptor and alpha (*Rxrα*). All primers were synthesized by Integrated DNA Technologies (Leuven, Belgium) and primer sequences are summarised in the Supporting information Table S1.

The following cycling conditions were used in CFX384 Touch real-time Detection Cycler (Bio-Rad): after initial activation for 30 s at 98 °C, the cycle steps of denaturation for 5 s at 95 °C and annealing/elongation for 20 s at 60 °C were repeated for 39 cycles. Melting curve analysis was applied to confirm the integrity of generated RT-qPCR products by a single amplicon peak. The melting curve was generated from 65 °C to 95 °C with an increment of 5 °C/5 min.

### 2.6 RT-qPCR for miR quantification

For miR expression profiling, we used the miScript SYBR Green PCR Kit (Qiagen, Hilden, Germany), as described by the supplier. The total volume of each reaction was 10 μl, including 1 μl template cDNA, 1 μl 10 × miScript Universal Primer as well as 1 μl of the specific 10 × miScript Primer Assay. The following conditions were used: initial activation 15 min at 95 °C, denaturation 15 s at 94 °C, annealing 30 s at 55 °C and extension 30 s at 70 °C. After data collection at the extension step, melting curve analysis was performed in the same manner as that mentioned for gene expression profiling. For normalisation, we calculated the geometrical average of all measured miRs, including miR-26b, miR-29a-5p, miR-29b-5p, miR-93, miR-96, miR-103, miR–miR-146, miR-221 and miR-365, as well as the miScript PCR Controls RNU5a, RNU6b, RNU1a, SNORD25 and SNORA73a [28]. Quantified miR sequences are listed in the Supporting information Table S2.

### 2.7 Expression data normalisation

All internal reference genes at the mRNA and miR level were evaluated with geNorm [29] and NormFinder [30] both part of the gene expression analysis software suite GenEx 5.4.2 (MultiD, Gothenburg, Sweden). All relative expression changes are represented as fold changes according to the following formula: *FC* *=* *2^−ΔΔCt^* [31].

### 2.8 MIQE compliance

Quantitative PCR assays were validated according to the MIQE guidelines [32] (for details see the MIQE checklist in the appendix). In brief; sample and RNA integrity were evaluated; exon spanning primer design was done using Primer 3 web version; PCR assay efficiency (96.2 ± 5.6%) was evaluated using dilution series; relative gene expression data were analysed with MIQE approved algorithms using GenEx 5.4.2 software package.

### 2.9 Microarray mRNA gene expression profiling

Using the GeneChip® Mouse Gene 1.0 ST Array (Affymetrix, Santa Clara, United States), 28,853 well annotated genes are detectable. Using the Gene Chip Array, the gene expression profiles in the presence and absence of 3 μM ATRA were analysed in 3T3-L1 cells at eight different time points (0, 2, 4, 7, 10, 24, 48 and 96 h). Therefore, three independent cell culture experiments were conducted, and RNA was extracted as mentioned above. In total, 100 ng total RNA of each sample was used as the starting material for microarray hybridisation [33]. An Affymetrix processing protocol was utilised [34], and 45 arrays were performed by the Genomic Core Facility of the European Molecular Biology Laboratory (EMBL, Heidelberg, Germany). For data normalisation, the GeneSpring GX Software (Agilent Technologies, Santa Clara, United States) [35] was used and the robust multichip analysis (RMA) algorithm was applied. The data were analysed with regard to the quality and quantity of the data. The microarray data were verified by RT-qPCR, and the Pearson correlation coefficients were calculated. Data were analysed with the Multiexperiment Viewer (Dana-Faber-Cancer-Institute, Boston, USA) [36]. Data were generated as well as analysed following the MIAME guidance [37].

### 2.10 MiR expression analysis

For miR expression profiling, the nCounter Mouse miR Expression Assay (NanoString, Seattle, United States) was used [38], [39]. Using this analysis system, 600 murine and murine-associated viral miRs are detectable. A digital colour-coded barcode technology was utilised, and each barcode was attached to a single target-specific probe corresponding to miR of interest. MiR-specific probes were designed against the annotations in mirBase version 18 [40], [41]. These target-specific probes were mixed together with controls; therefore, a multiplexed code set was formed [38], [39]. The miR expression in the presence and absence of 3 μM ATRA was analysed in two independent cell culture experiments at nine different time points (0, 2, 4, 7, 10, 24, 48 and 96 h and 288 h = 12 days). As a starting amount for the assays, 100 ng total RNA was analysed per sample. Each assay was normalised on the basis of the geometric mean of the top 100 expressed miRs, and a normalisation factor relative to the mean of all samples was calculated for each assay using the nSolver Analysis Software (NanoString Technologies). The normalisation factors for all assays ranged between 0.1 and 10; this range is also recommended by NanoString Technology itself. Heatmaps were generated using the Multiexperiment Viewer software [36].

### 2.11 Genomatix pathway system analysis (GePS)

For analysis of enriched biological processes (GO terms) in DMI-treated and DMI + 3 μM ATRA-treated cells, we utilised the GePS Software (Genomatix, Munich, Germany). Based on information from public and proprietary databases as well as co-citations in the literature, this software enables a comprehensive analysis of enriched GO terms [42].

### 2.12 Gene ontology analysis

The GO terms determined by GePS were analysed by the open source software REVIGO (Laboratory for information systems at the Rudjer Boskovic Institute, Zagreb, Croatia) [43]. REVIGO summarises lists of GO terms by excluding redundant terms using a clustering algorithm. Only non-redundant terms are visualised in clusters (rectangles). Related GO terms were summarised in superclusters with the same colour code. We selected the 50 most enriched GO terms among the ATRA regulated genes over time (0–96 h).

### 2.13 Building miR–mRNA regulatory networks

For building up an miR–mRNA regulatory network, we initially set up a prediction-based network by combining the conserved target predictions of TargetScan [44]. We then fitted a generalised linear model (GLM) on the scaled expression profiles of mRNA and its predicted miRs for each gene and computed the regularisation path for the elastic net penalty, which is implemented in the GLMnet package for R [45]. Using 10-fold cross-validation, we could select only those miRNAs that explain the corresponding mRNA regulation. Because we were primarily interested in anti-correlative effects of miR–mRNA interaction, we introduced a negativity constraint by modifying the implementation in the GLMnet package [45]. The weight of the miR–mRNA relation corresponds to the absolute coefficient of the respective miR in the GLM.

To identify pathways that are specifically affected by the network structure of the resulting miR–mRNA network, we first calculated a *p*-value of pathway over-representation for each gene in the network. From this, we defined a set of neighbour genes for each gene. This set includes all genes that are targeted by the same miRs as the gene itself. We then applied Fisher’s exact test to test for over-representation of the pathway gene set among the neighbour gene set of each gene. We obtained a distribution of *p*-values for every pathway. This was done for all pathways derived from the Kyoto Encyclopedia of Genes and Genomes (KEGG) [46]. Following this, we randomly permuted the labels 100 times and tested each time whether the *p*-value distribution of the original network was significantly shifted to lower *p*-values compared with the respective random network using a two-sample Kolmogorov–Smirnov test. The visualisation of the pathways and the node colouring according to the pathway over-representation was performed using Cytoscape 2.8.3 (Cytoscape Consortium, San Diego, USA) [47].

### 2.14 Oil-red-O-staining

Lipid accumulation in ATRA-treated and −untreated 3T3-L1 cells was detected by oil-red-O staining [48]. On day 12 (288 h) after starting differentiation, the medium was discarded and the cells were air dried for 20 min and fixed at room temperature by adding 10% formalin in PBS for 15 min. After fixation, the cells were air dried again and stained with a filtered oil-red-O working solution for 1 h on the orbital shaker. For the working solution, the 0.5% oil-red-O isopropanol stock solution was diluted 3:2 with water. Stained cells were washed three times with distilled water, the wells were air dried and the residual colour on the well sides was removed with an isopropanol-soaked cotton stick. The six-well-plates were scanned and stored at 4 °C until dye extraction. The dye was extracted from cells by adding 1 ml of the dye extraction solution. The cells were incubated with the solution for 1 h on the orbital shaker, and the absorption was measured at 492 nm with a plate reader (Tecan Deutschland GmbH, Crailsheim, Germany).

## 3 Results

### 3.1 ATRA inhibits adipogenesis in a time-dependent manner

**Fig. 1:**
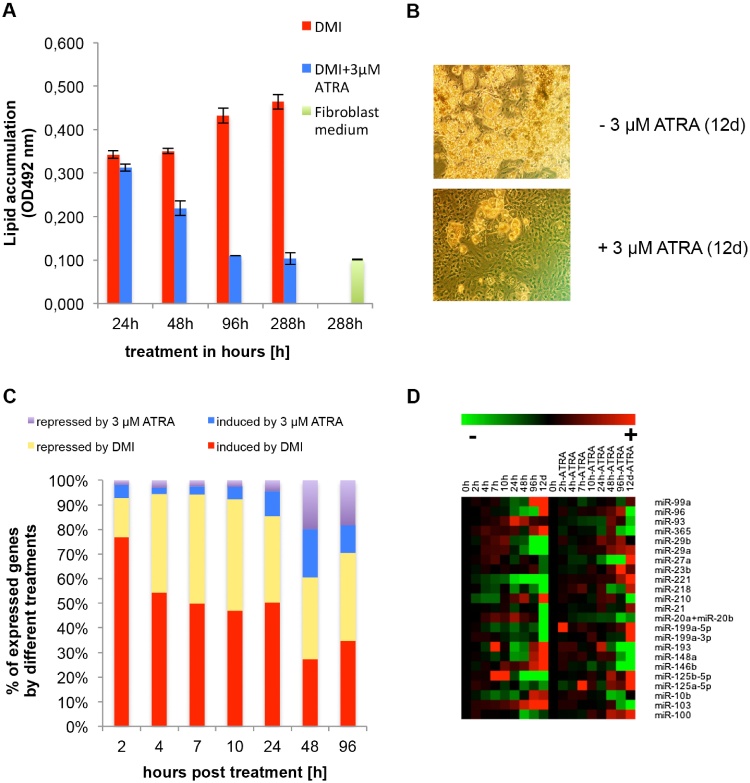
ATRA modulates the phenotype as well as the post-transcriptional mechanisms in 3T3-L1 cells. (A) Oil-red-O-staining of 3T3-L1 cells: Quantification of lipid accumulation in DMI-treated (control) and DMI + 3 μM ATRA-treated 3T3-L1 cells. After the indicated times, DMI (±3 μM ATRA) was replaced by growth medium without ATRA. Only in the labelled 96-h approach, DMI + 3 μM ATRA was changed to growth medium with ATRA for 288 h (12 d). (B) 3T3-L1 cell phenotypes: lipid accumulation in ATRA-treated and -untreated cells were visualised by microscopic images. (C) Relative gene expression trend, either up- or down-regulated, in the control and ATRA-treated cells in the time frame 2 h to 96 h (compared with 0 h). (D) MicroRNA expression profiling: Expression changes in ATRA-untreated 3T3-L1 cells are presented relative to 0 h, whereas the expression changes in ATRA-treated 3T3-L1 cells are presented relative to the corresponding untreated samples. An increase in microRNA expression is represented by accelerating red intensities, whereas decreasing ratios are represented by accelerating green intensities.

The well-known characteristic time-dependent phenotype changes were detected during differentiation of 3T3-L1 cells, as assessed by oil-red-O staining (Fig. 1**a**). The cells were cultured in triplicates for 24, 48 and 96 h in DMI medium (±3 μM ATRA), and DMI medium was changed to growth medium (±3 μM ATRA) after the indicated times. All cultured cells were stained after 12 days (288 h) of differentiation. The growth medium was renewed every 2 days. Cultures exposed only for the first 24 h to 3 μM ATRA showed no significant inhibition of differentiation. Lipid accumulation was similar to control cultures that were exposed for 24 h to DMI only. The exposure to 3 μM ATRA for 48 h resulted in the suppression of lipid accumulation by 40%. Incubation of the cells with ATRA for more than 96 h caused further inhibition of lipid accumulation. Therefore, incubation of 3T3-L1 preadipocytes with 3 μM ATRA for 288 h (12 days) was highly efficient; lipid droplets were only accumulated in a few cells. The capability for differentiation in cells treated with ATRA for a long term (ATRA treatment persists from 0 h to 288 h) was similar to the phenotype of undifferentiated cells, which were cultivated after growth arrest for a further 288 h (12 days) with fibroblast medium (Fig. 1a). The morphology of control and ATRA-treated cells was also visualised microscopically as shown in Fig. 1**b.** In ATRA-treated cultures, only a few mature adipocytes were detectable, whereas the control cultures were fully differentiated.

### 3.2 High-throughput gene expression profiling in ATRA-treated and -untreated cells

Using microarray technology, gene expression in DMI-treated and DMI + 3 μM ATRA-treated 3T3-L1 cells was analysed during the first 96 h. The squared correlation coefficients for all corresponding triplicates were calculated (r^2^ > 0.95). Only transcripts with significant Affymetrix signal values (>30) and with a twofold up- or down-regulation at a minimum of one time point were included in the analysis. Fig. 1**c** depicts an overview of the relative gene expression trend, either up- or down-regulated, in the control and ATRA-treated cells. After 2 h, 388 transcripts were significantly differentially expressed in 3T3-L1 preadipocytes. Of these, 298 transcripts (77%) were up-regulated by DMI and 62 transcripts (16%) were down-regulated by DMI, whereas only 21 transcripts (5%) were induced by 3 μM ATRA and only seven transcripts (2%) were repressed by 3 μM ATRA. After 96 h, 1314 transcripts were significantly regulated in 3T3-L1 preadipocytes. In total, 455 transcripts (35%) were up-regulated by DMI and 471 (36%) were down-regulated by DMI. Finally, 148 transcripts (11%) were induced by 3 μM ATRA and 240 transcripts (18%) were repressed by ATRA.

### 3.3 High-throughput miR expression profiling in ATRA-treated and -untreated cells

Under the same culture conditions, miR expression in ATRA-treated and -untreated cells was analysed in two independent experiments. First, the quality of miR expression data was evaluated, including calculation of an average squared correlation coefficient r^2^ = 0.88 of the corresponding duplicates and using a two-way analysis of variance (ANOVA) (critical *p*-value for significance: 0.01) to select significantly regulated miRs. Furthermore, only miRs exhibiting stable NanoString signal values (>50) at all time points and with a twofold up- or down-regulation in at least one time point were selected for further analysis. There were 23 miRs that fulfilled these criteria (Fig. 1**d**; the expression values are presented in Table 1, Table 2. These 23 selected miRs were used for the generation of the miR–mRNA network. As proof of the integrity of our miR expression assay data, the expression levels of three putative adipogenesis-relevant miRs (miR-103, miR-146 and miR-221) and five highly regulated miRs (miR-29a, miR29b, miR-365, miR93 and miR96) were analysed by RT-qPCR (S1 and S2 Figures; the corresponding expression values are summarised in the Supporting information S3 and S4 Tables). The Pearson correlation coefficient between the nCounter miR expression assay data and the RT-qPCR was highly significant (r = 0.80) (S3a Figure). For the calculation, we used the expression values of the above mentioned miRs out of the miR expression assays (Table 1, Table 2) as well as the corresponding expression values of the RT-qPCR evaluation (see the Supporting information S3 and S4 Tables).

### 3.4 Inhibition of fat cell differentiation by ATRA involves the repression of adipogenic specific transcription factors

**Fig. 2:**
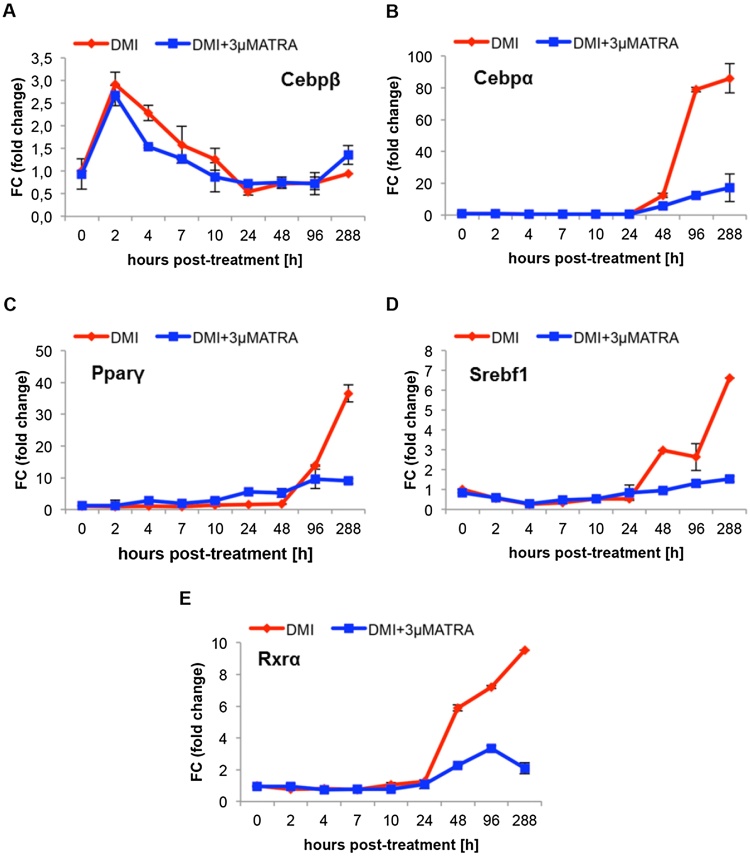
Quantitative analysis of mRNA expression by RT-qPCR of adipogenetic specific regulators in DMI-treated (red) and DMI + 3 μM ATRA-treated (blue) preadipocytes in a time course study (0–288 h). ATRA has no effect on the gene expression of (A) Cebpβ, whereas the expression of (B) Cebpα (C) Pparγ (D) Srebf1 (E) Rxrα is inhibited by ATRA. Expression changes are presented relative to 0 h.

For microarray mRNA data validation, we quantified the expression profiles of adipogenesis-specific transcription factors such as *Cebpβ, Cebpα, Pparγ, Srebf1* and *Rxrα* (Fig. 2a–e). The expression changes are presented relative to baseline. Compared with the expression profile of *Cebpβ* in DMI-treated cells ATRA has no influence on *Cebpβ* expression after 2 h (Fig. 2a). At 288 h (12 days) post-induction, *Cebpα* (FC = 86) (Fig. 2b), *Pparγ* (FC = 37) (Fig. 2c), *Srebf1* (FC = 7) (Fig. 2d) as well as *Rxrα* (FC = 10) (Fig. 2e) was induced by DMI relative to 0 h, whereas the induction in ATRA-treated cells was downregulated (*Cebpα*; FC = 17, *Pparγ*; FC = 9, *Srebf1*; FC = 2, *Rxrα*; FC = 2). The microarray gene expression data were confirmed by RT-qPCR, and we also calculated the Pearson correlation coefficient between the microarray data and the RT-qPCR data (r = 0.7) (S3b Figure). For the calculation of the Pearson correlation coefficient, we used the expression values of the above mentioned genes from the microarray experiments as well as the expression values of the RT-qPCR evaluation (S5–S8 Tables).

### 3.5 Regulation of biological processes in ATRA-treated 3T3-L1 cells

**Fig. 3:**
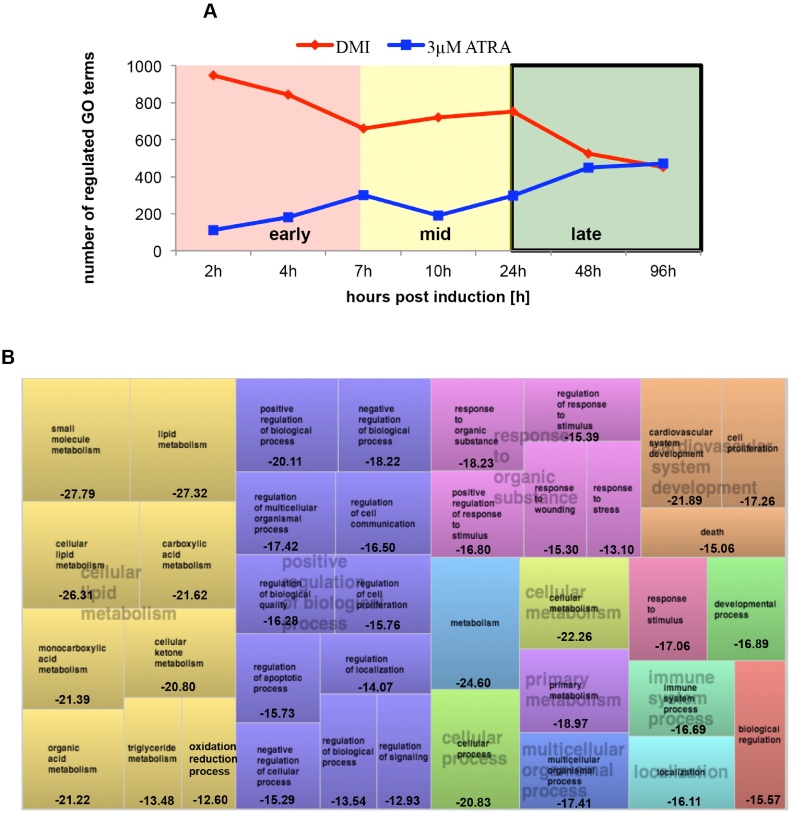
Regulation of biological processes in DMI-treated and DMI + 3 μM ATRA-treated 3T3-L1 cells. (A) Regulated GO terms in DMI and ATRA treated 3T3-L1 cells in early stages (2, 4 and 7 h post-induction) and mid stages of differentiation (10 and 24 h), ATRA (blue) only has small effects on biological processes compared with DMI treatment (red). (B) Cluster of ATRA regulated GO terms in the late stages of early differentiation (48/96 h post-induction). GO terms were analysed by the Genomatix Software and the top 50 regulated GO terms per time were selected and visualised with Revigo. The late regulation (48 and 96 h) of GO terms is visualised. The cellular lipid metabolism is most affected by ATRA, shown in the left yellow supercluster. The size of the rectangles represents the level of significance, whereby the log10 p-values of the GO term enrichments are given at the bottom.

We next examined the effects of treatment and exposure time (0–96 h) to 3 μM ATRA on biological processes in 3T3-L1 preadipocytes. An overview of the impact of DMI as well as of ATRA exposure on the regulation of biological processes is presented in Fig. 3a. The effects of DMI on biological processes during the early stages (2, 4 and 7 h post-induction) and the mid stages (10 and 24 h post-induction) were stronger than the regulatory effects of ATRA. In the late stages (48 and 96 h post-induction) of early differentiation, the regulation of GO terms by DMI and ATRA was almost identical. In summary, at 2 h post-induction, 949 GO terms were significantly regulated by DMI treatment, whereas only 112 GO terms were regulated by ATRA treatment. The regulatory effects mediated by ATRA gradually increased, and in the late stage of early differentiation (48 and 96 h post-induction) the regulatory effects of DMI and ATRA were similar: 451 GO terms were subjected to treatment with DMI and even 471 GO terms were controlled by ATRA (Fig. 3a).

The most affected superclusters in early-stage differentiation were cell proliferation (blue rectangle) and tissue development (red rectangle) (S4a Figure). In mid-stage differentiation, the most affected superclusters were multicellular organism process (dark purple rectangle), response to wounding (light purple rectangles) and cell proliferation (yellow rectangles) (S4b Figure). In the late stages of differentiation (48/96 h post-induction), the most affected supercluster upon ATRA was found to be cellular lipid metabolism (Fig. 3b).

### 3.6 The metabolism of 3T3-L1 cells is regulated by ATRA

**Fig. 4:**
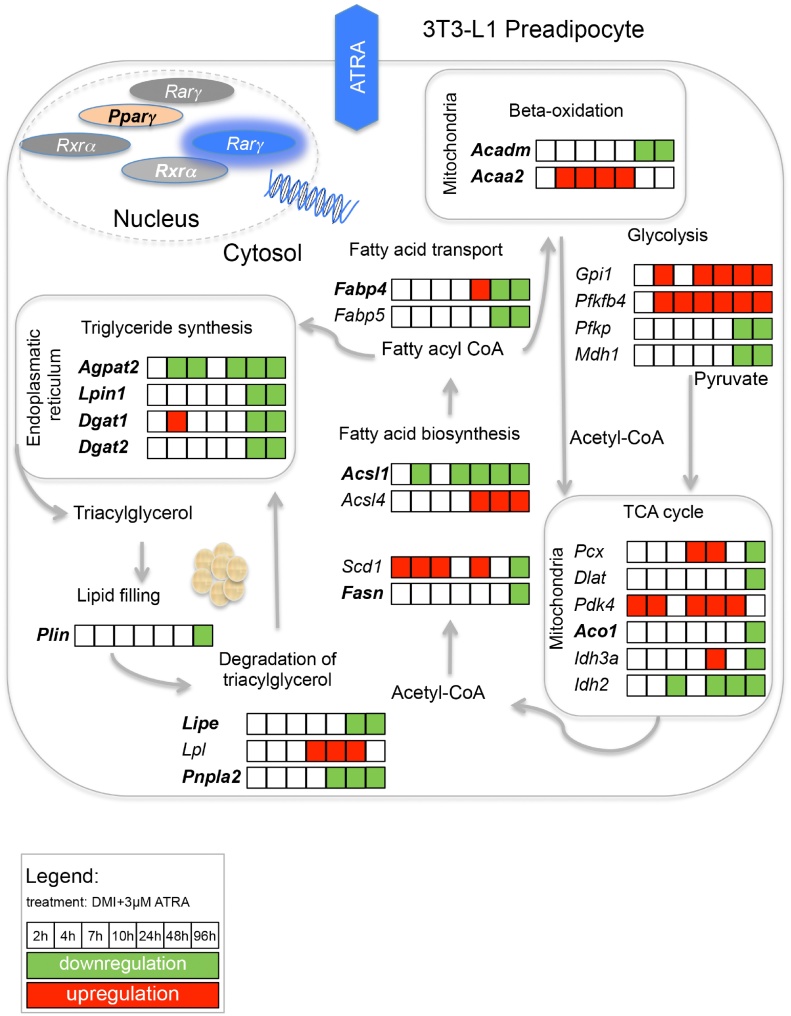
Schematic overview of metabolic processes that are affected by ATRA during adipogenesis in 3T3-L1 cells over 96 h. The highlighted genes are direct targets of *Pparγ*. Gene expression changes in ATRA-treated 3T3-L1 cells are presented relative to the corresponding untreated samples. The colour code reflects an up- or down-regulation greater than 1.5.

In the next step, we analysed mRNA gene expression of key regulators within energy metabolism. The gene expression related to triglyceride synthesis in 3T3-L1 cells was negatively influenced by ATRA. In detail, the genes that were involved in beta-oxidation, the tricarboxylic acid cycle (TCA cycle), fatty acid biosynthesis, fatty acid transport, triglyceride synthesis, lipid accumulation as well as degradation of triacylglycerol for energy production were down-regulated by ATRA (Table 3; Fig. 4). In this context, the mRNA expression of acetyl-Co enzyme A dehydrogenase (*Acadm*), acetyl-CoA acyltransferase 2 (*Acaa2*), phosphofructokinase (*Pfkp*), malate dehydrogenase 1 (*Mdh1*) and pyruvate carboxylase (*Pcx*) was inhibited by ATRA in the late stage of differentiation. Isocitrate dehydrogenase 2 (*Idh2*) was down-regulated by ATRA after 24 h. The transcription of fatty acid synthase (*Fasn*) as well as fatty acid-binding proteins 4 and 5 (*Fabp4; Fabp5*) was inhibited by ATRA. Triglyceride synthesis as well as lipid accumulation involves the expression of 1-acylglycerol-3-phosphate O-acyltransferase *2* (*Agpat2*), lipin 1 (*Lpin1*), diacylglycerol O-acyltransferase 2 (*Dgat2*) and perilipin (*Plin*) that were also inhibited by ATRA. In addition, the gene expression pattern subjected to the degradation of triacylglycerol was negatively regulated by ATRA; the expression of hormone-sensitive lipase (*Lipe*) and lipoprotein lipase (*Lpl*) was also suppressed after 96 h.

### 3.7 The regulation of biological pathways in ATRA-treated 3T3-L1 preadipocytes relative to the observed miR-mRNA interactions

**Fig. 5:**
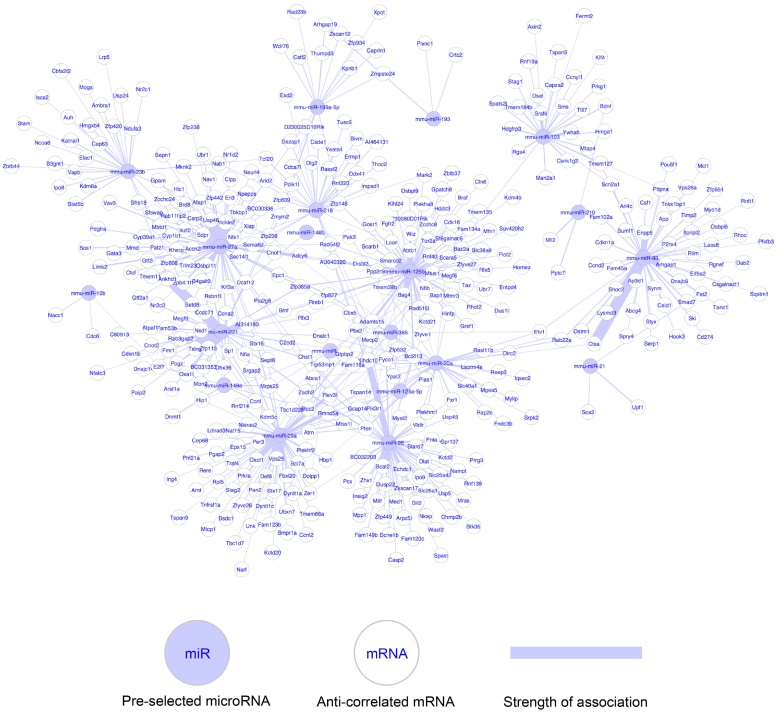
Joint analysis of real microRNA and mRNA data on the basis of target prediction of TargetScan Mouse 5.2 [44] and a linear regression model [49]. The edges in the network correspond to an association of the microRNA to the gene sequence and its expression level. The edge weights denote the anti-correlation (=the negative coefficient of the respective microRNA-mRNA relation in the multiple regression model).

**Fig. 6:**
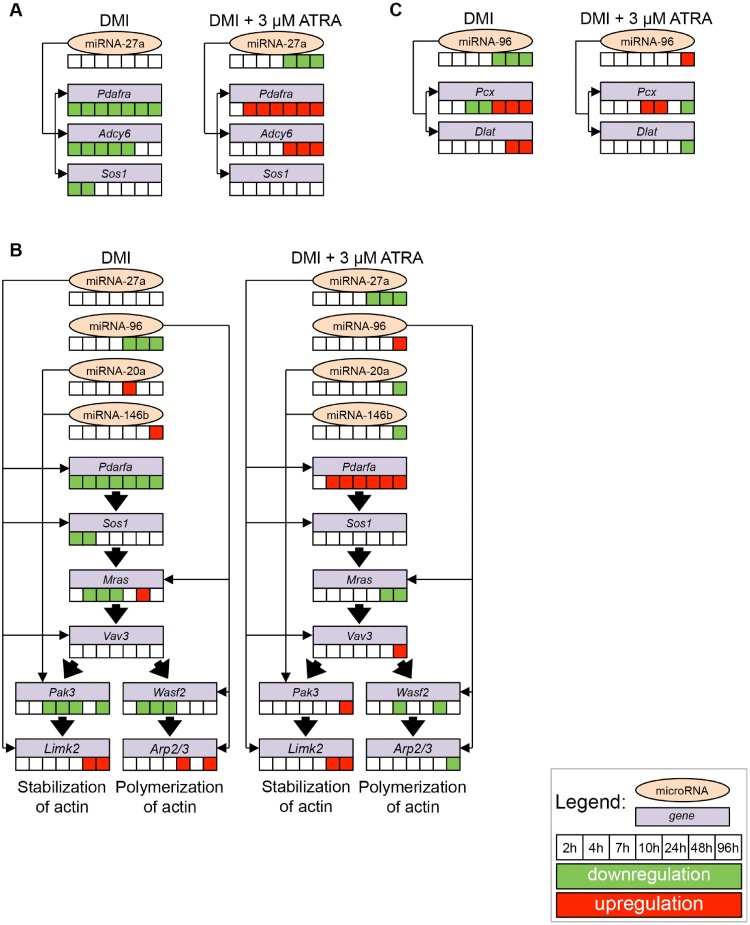
Overview of the most significantly regulated KEGG pathways in ATRA-treated 3T3-L1 cells after *in silico*-based joint analysis of microRNA and mRNA data with the corresponding expression data over time and treatment. Expression changes of the ATRA-treated 3T3-L1 cells are presented relative to the corresponding untreated samples. The colour code reflects a regulation greater than 1.5. (A) Gap junction, (B) regulation of actin cytoskeleton, (C) TCA cycle. (For the whole network, see electronic supplementary S5–S7 Figures).

Using a multiple linear regression model [49] combined with the derived miR and mRNA expression data and the target predictions of TargetScan Mouse 5.2 [44], [50], an *in silico* based miR–mRNA network was generated (Fig. 5). Fig. 6 summarises the miR–mRNA interactions with specific pathway annotations and the corresponding expression profiles detected by our high-throughput transcriptomic data screening (Table 1, Table 2, Table 3, Table 4). The comprehensive miR–mRNA networks are visualised in the Supporting information S5–S7 Figures. Using our *in silico* based pathway-expression analysis tools, we were able to show the following: 1) The most affected pathways subjected to our data sets were gap junction signalling (functional locality score = 2.86E-06), the rearrangement of the actin cytoskeleton (functional locality score = 1.06E-10) and the regulation of the TCA cycle (functional locality score = 5.84E-26). 2) MiR-27a and miR-96 are probably the most important co-actors in these pathways, in which miR-27a is inhibited by ATRA and miR-96 is induced by ATRA. 3) Using *in silico*-based tools, we were also able to visualise the interactions of miRs with mRNAs with specific pathway annotation (mRNAs are visualised as rectangles in the Supporting information S5–S7 Figures) subjected to our own expression data sets. Therefore, *in silico*, we could visualise the predicted interactions of miR-27a and the corresponding transcripts such as the platelet-derived growth factor receptor, alpha polypeptide (*Pdgfra*), son of sevenless 1 (*Sos1*), vav 3 oncogene (*Vav3*) and LIM kinase 2 (*Limk2*). All mentioned genes are associated with gap junction signalling on the basis of the information of KEGG pathways (KEGG: mmu04540) (Fig. 6a) as well as the rearrangement of the cytoskeleton (Fig. 6b) (KEGG-Pathway: mmu04810). Furthermore, we visualised the interaction of miR-96 with the predicted transcripts of the WAS protein family, member 2 (*Wasf2*), the actin related protein 2/3 complex (*Arp2/3*) [51] and the muscle and microspike RAS (*Mras*). All these mentioned genes are also directly involved in cytoskeleton regulation (KEGG-Pathway: mmu04810). In addition to the potential regulatory effects of miR-96 inside the actin cytoskeleton (Fig. 6b), *Pcx* and dihydrolipoamide S-acetyltransferase, the E2 component of the pyruvate dehydrogenase complex (*Dlat*), which are key regulators of the TCA cycle (KEGG-Pathway: mmu00020) [52] are forecasted as targets of miR-96 by TargetScan Mouse (Fig. 6c).

## 4 Discussion

There is increasing evidence that miRs play a very important role in metabolism and energy homeostasis. Therefore, this regulatory small RNA species may be involved in the pathogenesis of disorders such as the metabolic syndrome or type 2 diabetes. In this study, we used large-scale transcriptomic methods to profile the global expression of miRs and mRNAs in 3T3-L1 preadipocytes in a high-resolution time frame. We combined these data to generate global molecular networks in order to explain the phenotype of ATRA-treated 3T3-L1 cells (Fig. 1b).

It is known from the literature [5], [10], [11] that 3T3-L1 cells are very sensitive to ATRA exposure during the early stages of differentiation; however, simultaneously, the ATRA-dependent inhibition is reversible within the first 48 h [11]. This may imply that the longer the incubation time with ATRA, the stronger are the inhibitory effects of ATRA on fat cell differentiation. We could confirm this effect on phenotypic development (Fig. 1a and b). Moreover, these results are in accordance with our transcriptomic data (Figs. 1c and Fig. 2).

In our experiments, a modulation of miR expression in DMI as well as in ATRA-treated cells was observed after 24 h but not at earlier stages of differentiation, suggesting that miRs modulate adipocyte differentiation after induction of this program (Fig. 1d). Similar time-dependent miR expression patterns for DMI-induced differentiation of 3T3-L1 cells have been reported by Kajimoto et al. [53] for the TPA-induced differentiation of HL-60 cells [54] as well as for the neuronal differentiation of primary rat cortical cells [55]. Moreover, our miR expression data (Fig. 1d) confirm the findings of Xie (2009) [56] and Knelangen [57] that miR-221 and miR-125b-5p are down-regulated during differentiation of 3T3-L1 cells, whereas miR-103 and miR-146b are up-regulated. To our knowledge, our study is the first detailed report on the complex modulation of miR expression in 3T3-L1 preadipocytes by ATRA.

Similar to the mRNA and miR expression pattern, no adipocyte-specific GO terms were enriched in ATRA-treated cells during the early and mid stages of differentiation (see the Supporting information S4a and S4b Figs.). A possible explanation for this finding is that on one hand, it was shown that the mitotic clonal expansion is not affected by ATRA [16], [58], while on the other hand, it is known and confirmed by our data that the inhibitory effects of ATRA are reversible during the first 48 h of differentiation (Fig. 1a and b) [11].

In Fig. 4, the mRNA-based regulation of metabolism in ATRA-treated cells is summarised. We could show that in the presence of ATRA, transcripts encoding for important metabolic enzymes and kinases related to energy production are down-regulated. For example, genes involved in fatty acid biosynthesis were suppressed by ATRA exposure. The gene activity of *Fasn* [59], an enzyme that catalyses the synthesis of long chain fatty acids, was inhibited. Perilipin (*Plin*) is a protein that is located at the surface of lipid droplets and controls both lipid storage and activation of lipolysis [60], [61], [62]. In ATRA-treated cells, no lipids were accumulated for storage or lipolysis (Fig. 1b), and one possible explanation is that the mRNA expression of *Plin* was down-regulated by ATRA after 96 h; therefore, the protein production of *Plin* is possibly inhibited.

The TCA cycle is a central metabolic pathway [63] that combines the anabolism as well as catabolism of biomolecules such as amino acids, monosaccharides and fatty acids [64], [65]. Rate-limiting steps in metabolism are normally key positions for regulation, such as starting points for other pathways. Newton et al. [2011] showed that developing preadipocytes enter a state of metabolic-overdrive, where the activity of the TCA cycle is increased [66]. Our results indicate that ATRA can mitigate this exaggerated induction at the level of transcription. For example, the expression of *Pdha1,* which catalyses with *Pcx* the pyruvate entry as acetyl-CoA in the TCA, can be inhibited by pyruvate dehydrogenase kinase, isoenzyme 4 (*Pdk4*), which is located in the mitochondrial matrix [67]. Attia et al. showed that the transcript of *Pdk4* is up-regulated by ATRA [68]. We could also demonstrate that the gene expression of *Pdk4* was induced after 2, 4, 10, 24 and 48 h by ATRA.

Post-transcriptional regulation of transcription by miRs is a very important process for metabolic processes and thereby for later phenotype formation. Hence, analysis of the mRNA–miR interplay followed by signal transduction is a crucial aspect with regard to the regulation of metabolic pathways. For the communication between adjoined cells, gap junctions are required. Communication by gap junctions is very sensitive to a multitude of stimuli including changes in the concentration of intracellular Ca^2+^, cAMP, IP3 and phosphorylation/dephosphorylation processes mediated by kinases [69]. In Fig. 6a, the miR–mRNA-dependent regulation of gap junction signalling is shown. From the target predictions of TargetScan Mouse and our *in silico* based analysis, we know that miR-27a exhibits binding sites for *Pdgfra*, a receptor tyrosine kinase that is a known suppressor of adipogenesis and *Pparγ* activity [70], [71]. *Pdgfra* is enriched in non-adipogenic cells and is able to mediate anti-adipogenic activities in 3T3-L1 cells [70]. Therefore, anti-adipogenic functions could be mediated in 3T3-L1 cells if *Pdgfra* is activated. During fat cell differentiation, the gene expression of *Pdgfra* is down-regulated; therefore, the inhibition of connexin communication between cells may occur. Hence, the *Pdgfra*-mediated anti-adipogenic signal cascade is inhibited during adipogenesis [72]. Down-regulation of miR-27a by ATRA may cause the induction of *Pdgfra* and the activation of the anti-adipogenetic signal cascade.

The rearrangement of the cytoskeleton is essential for cell shape, cell mobility as well as intracellular transport [73]. This rearrangement depends on a highly sophisticated signalling cascade, including actin depolymerisation and rearrangement as well as actin filament reorganisation. We showed that miR-96 is a miR that is associated with the rearrangement of the cytoskeleton (Fig. 6b) through targeting genes with pathway-specific annotations (rectangle labelled genes in the Supporting information S6 Fig.). Laine et al. have already shown the importance of miR-96 for different developing processes in human mesenchymal stromal cells [74]. Interestingly, on day 12 (288 h), the regulation of miR-96 was inverse compared with the expression profile at earlier time points; DMI induced and ATRA repressed the expression of miR-96 (Figs. 1d and 6b). Recently, Xie et al. showed that miRs that are induced during adipogenesis tend to be down-regulated in the obese state and vice versa [56]. Our explanation for the inverse expression of miR-96 is that in DMI-treated cells, miR-96 is repressed during differentiation and the pro-adipogenic signalling cascade is switched on. After 288 h (12 days), lipid filling is completed and transcription factors for terminal differentiation have to be switched off. For this purpose, an induction of miR-96 is required. MiR-96 is induced by ATRA after 96 h; therefore, pro-adipogenic factors are inhibited and lipogenesis is suppressed. In addition, *Arp2/3 and Wasf2* build the WAVE complex [75], which also takes part in actin filament reorganisation, because the gene product of *Wasf2* can mediate the association of receptor kinases and actin [76], [77], [78]. Interestingly, *Arp2/*3 is forecasted as a further target of miR-96 by TargetScan Mouse. Therefore, the miR-96–*Arp2/3* interaction may be a central step in the regulation of the rearrangement of the actin cytoskeleton in 3T3-L1 preadipocytes and in the conversion of 3T3-L1 cells from a fibroblast-like to mature adipocyte-like morphology. To confirm our hypothesis, further studies, including knockdown experiments as well as overexpression experiments, are necessary.

## 5 Conclusion

In summary, in this comprehensive study of high-throughput data, we generated a global miR–mRNA interaction network to explain physiological and morphological changes modulated by ATRA in 3T3-L1 pre-adipocytes. We could show that the cellular lipid metabolism is negatively affected by ATRA. Furthermore, we were able to demonstrate that miR-27a and/or miR-96 are very important regulators and gap junction signalling, the rearrangement of the actin cytoskeleton as well as the citric acid cycle are the most affected pathways with regard to inhibitory effects of ATRA in 3T3-L1 preadipocytes. The experimental workflow and the integrative microRNA–mRNA data analysis shown in this study represents a possibility for illustrating interactions in highly orchestrated biological processes. On the other hand, this model of data analysis may also be used for the pre-selection of potential new biomarkers with regard to obesity or for the identification of new pharmaceutical targets.

## Conflict of interest

All authors disclose there is no actual or potential conflict of interest including any financial, personal or other relationships with other people or organizations.

## Authorship

Katharina Stoecker: conception and design of the study, data analysis and interpretation, drafting the manuscript, final approval.

Steffen Sass: integrative data analysis, data interpretation, drafting the manuscript.

Fabian J. Theis: integrative data analysis, data interpretation, drafting the manuscript.

Hans Hauner: data analysis, data interpretation, drafting the manuscript, final approval.

Michael W. Pfaffl: conception and design of the study, data analysis and interpretation, drafting the manuscript, final approval and submission.

## Figures and Tables

**Table 1:** Changes in microRNA expression in DMI-treated 3T3-L1 cells.

microRNA	0 h DMI	2 h DMI	4 h DMI	7 h DMI	10 h DMI	24 h DMI	48 h DMI	96 h DMI	12d DMI
miR-99a	0	0,1	0,1	0,1	−0,1	−0,4	−0,3	1,1	1,5
miR-96	0	0	0,2	0	−0,1	−0,4	−0,7	−0,8	2,6
miR-93	0	0	0,1	0,2	0,3	0,9	0,6	0,2	0,2
miR-365	0	0,2	0,3	0,2	0,2	0,3	−0,1	0,6	2,3
miR-29b	0	−0,1	0,2	0,3	0,4	−0,3	−0,3	−1,1	−2
miR-29a	0	0	0,3	0,3	0,3	−0,1	−0,5	−1,1	−1,9
miR-27a	0	0,1	0,2	0,3	0	−0,2	0,2	0	−0,4
miR-23b	0	0	0,1	0	0,1	0,1	0,1	0,3	−0,4
miR-221	0	−0,1	−0,2	−0,3	−0,4	−0,9	−1,7	−1,7	−2,7
miR-218	0	0	−0,2	−0,3	0,1	−0,5	−0,1	−0,5	−1,5
miR-210	0	−0,3	−0,4	−0,4	0,1	0,3	0,6	−0,1	2,4
miR-21	0	0	0,1	0	0,1	−0,2	0	0	−1,6
miR-20a/20b	0	0	0,2	0,4	0,3	0,6	0,3	0,1	−1,1
miR-199a-5p	0	0,1	−0,1	−0,3	−0,2	−0,4	−0,1	−0,3	−1,3
miR-199a-3p	0	0,1	0,2	0,1	−0,1	−0,2	−0,2	−0,1	−1,2
miR-193	0	0	−0,2	0	0,1	−0,7	−0,5	0,6	2,3
miR-148a	0	0,1	0	−0,2	0	−0,6	−0,5	0,7	2,9
miR-146b	0	−0,1	0,1	0	0,4	0,2	0,4	0,8	1,3
miR-125b-5p	0	0,1	0,1	0	0	−0,2	−1	−1	−1,4
miR-125a-5p	0	0,2	0,1	0	−0,2	0	−0,4	−0,2	−0,6
miR-10b	0	−0,1	−0,2	−0,4	−0,3	−0,5	0	0,8	0,7
miR-103	0	0,2	0,2	0,2	0,3	0,4	0,8	1,8	3,2
miR-100	0	0,2	0	0,1	0	0	−0,7	−0,5	−0,2

Fold changes are presented relative to 0 h (log_2_-transformed ratios).

**Table 2:** Changes in microRNA expression in 3 μM ATRA-treated 3T3-L1 cells.

microRNA	0 h ATRA	2 h ATRA	4 h ATRA	7 h ATRA	10 h ATRA	24 h ATRA	48 h ATRA	96 h ATRA	12d ATRA
miR-99a	0	0	0	−0,2	−0,1	0	0,1	−0,3	0,4
miR-96	0	0,2	−0,1	0,1	0,1	0,2	0,3	0,7	−2,8
miR-93	0	0	−0,1	−0,2	−0,2	−0,3	0,4	0,2	−0,8
miR-365	0	−0,1	0,1	0	0	−0,3	0,3	−0,7	−2,1
miR-29b	0	0,2	0	0,1	0,1	0,5	0,3	0,6	0
miR-29a	0	0	−0,1	0,1	0,2	0,2	0,5	0,6	0,7
miR-27a	0	0,1	−0,2	−0,2	0	−0,5	−0,9	−1	0
miR-23b	0	0	−0,1	0	0,2	0	0,3	0	0,4
miR-221	0	0,1	0,1	0,1	0,1	0,2	0,2	0,6	1,6
miR-218	0	0,1	0,2	0,3	0	0,3	−0,6	−0,4	0,7
miR-210	0	0,1	0,1	0	−0,1	0,3	0,4	−0,1	−2,9
miR-21	0	0	−0,1	0	0	0,2	−0,1	−0,1	0,5
miR-20a/20b	0	0	−0,1	−0,2	−0,2	−0,3	−0,2	−0,8	−0,5
miR-199a-5p	0	0	0,1	0,2	0,1	0	0,1	0,3	1,1
miR-199a-3p	0	0	0	0	0,3	0	−0,1	−0,1	1,1
miR-193	0	0,3	0,3	0	0,1	0,7	−0,4	−1,2	−2,3
miR-148a	0	0,2	0,1	0,2	0	0,3	0,2	−0,6	−2,4
miR-146b	0	0,2	−0,1	0	−0,3	−0,1	−0,2	−1,1	−2,1
miR-125b-5p	0	0	0	0	0,2	0	0,6	0,2	1
miR-125a-5p	0	−0,2	−0,1	0	0,2	−0,2	0,5	0,4	1
miR-10b	0	0,1	0,2	0,2	0	0	−0,7	−0,6	0,1
miR-103	0	0,2	0,1	0	0	0	0,2	−0,6	−1,6
miR-100	0	0	0	−0,1	0,3	0,1	0,7	0,6	0,9

Fold changes are presented relative to the corresponding untreated samples (log_2_-transformed ratio).

**Table 3:** Changes in mRNA expression in ATRA-treated 3T3-L1 cells.

Gene name	Gene description	2h	4h	7h	10h	24h	48h	96h
*Acadm*	acyl-Coenzyme A dehydrogenase, medium chain	0,1	0,3	−0,1	0,1	−0,3	−1,1	−1,4
*Acaa2*	acetyl-Coenzyme A acyltransferase 2	0,2	0,6	1	0,9	0,7	0,3	−0,3
*Pfkfb4*	6-phosphofructo-2-kinase/fructose-2,6-biphosphatase 4	0,5	1	1,2	0,9	1,2	0,7	0,8
*Pfkp*	phosphofructokinase, platelet	0	−0,1	−0,2	−0,3	−0,1	−1,4	−1,8
*Gpi1*	glucose phosphate isomerase 1	0,4	0,7	0,7	0,5	0,9	1	0,8
*Mdh1*	malate dehydrogenase 1, NAD (soluble)	−0,1	0,2	0	0,1	0,1	−0,4	−1,2
*Pcx*	pyruvate carboxylase	0,1	0,2	0	0,8	1	0	−0,5
*Dlat*	dihydrolipoamide S-acetyltransferase (E2 component of pyruvate dehydrogenase complex)	0	0	0	0,1	0,1	−0,3	−0,9
*Pdk4*	pyruvate dehydrogenase kinase, isoenzyme 4	1	1,1	0,2	1,4	0,6	0,6	−0,2
*Aco1*	aconitase 1	0,1	0,1	0,1	0	−0,1	−0,3	−1,2
*Idh3a*	isocitrate dehydrogenase 3 (NAD+) alpha	0,1	0,2	0,1	0	0,7	0,1	−1,3
*Idh2*	isocitrate dehydrogenase 2 (NADP+), mitochondrial	0	0	−0,4	−0,1	−0,6	−1,2	−0,6
*Fasn*	fatty acid synthase	0,1	−0,1	0	0	0,1	−0,3	−1,3
*Scd1*	stearoyl-Coenzyme A desaturase 1	1,2	1,3	1	0,5	1,5	0,2	−0,8
*Acsl1*	acyl-CoA synthetase long-chain family member 1	0	−0,4	−0,2	−0,4	−0,4	−2,5	−3,3
*Acsl4*	acyl-CoA synthetase long-chain family member 4	0,4	0	0,4	0	0,6	1,1	1,6
*Fabp4*	fatty acid binding protein 4, adipocyte	−0,1	0	0,5	0,3	0,8	−0,6	−1,3
*Fabp5*	fatty acid binding protein 5, epidermal	−0,3	0	0,2	0,4	0,5	−0,5	−1,3
*Agpat2*	1-acylglycerol-3-phosphate *O*-acyltransferase 2	0,1	−0,4	−0,4	−0,3	−0,7	−1,7	−2,9
*Lpin1*	lipin 1	0	0,3	−0,2	0	−0,1	−1,3	−1,8
*Dgat1*	diacylglycerol *O*-acyltransferase 1	0,5	0,9	0,4	0,1	−0,1	−1,7	−2,4
*Dgat2*	diacylglycerol *O*-acyltransferase 2	0	0,1	0,1	0	−0,2	−1,6	−2,5
*Plin*	perilipin	−0,1	0	0,1	0	0,1	0,1	−1,4
*Lipe*	lipase, hormone sensitive	0,1	0,2	−0,3	0	−0,2	−1,5	−2,6
*Lpl*	lipoprotein lipase	0	−0,1	0,1	0,5	1,1	0,7	−0,2
*Pnpla2*	patatin-like phospholipase domain containing 2	0,1	0	−0,1	0,1	−0,5	−2,7	−2,5
*Pdgfra*	platelet derived growth factor receptor, alpha polypeptide	0,3	1,3	1	1,5	1,5	2	2,1
*Adcy6*	adenylate cyclase 6	0,2	0,3	0	0,3	0,6	0,7	0,9
*Sos1*	Son of sevenless homolog 1 (Drosophila)	0,1	0	0,2	0,1	0,1	0,4	0,3
*Mras*	muscle and microspikes RAS	0	−0,1	−0,2	0,1	−0,1	−0,9	−0,7
*Vav3*	vav 3 oncogene	0,1	−0,1	0,1	0,3	0,1	0,2	0,7
*Pak3*	p21 (CDKN1A)-activated kinase 3	0	−0,3	−0,2	0,2	0,1	0,2	1
*Wasf2*	WAS protein family, member 2	0	−0,2	−0,4	0,1	−0,1	−0,4	−0,2
*Limk2*	LIM motif-containing protein kinase 2	0,2	0,4	0,2	0,1	0,4	1,1	0,7
*Arp2/3*	Actin related protein 2/3	0	0	−0,2	−0,1	−0,2	−0,1	−0,5

Fold changes are presented relative to the corresponding untreated samples (log_2_-transformed ratios).

**Table 4:** Changes in mRNA expression in DMI-treated 3T3-L1 cells.

Gene name	Gene description	2h	4h	7h	10h	24h	48h	96h
*Pcx*	pyruvate carboxylase	−0,1	−0,2	−0,4	−0,4	0,8	2,6	3,2
*Dlat*	dihydrolipoamide *S*-acetyltransferase (E2 component of pyruvate dehydrogenase complex)	0	0	0	−0,1	0,4	0,7	1,5
*Pdgfra*	platelet derived growth factor receptor, alpha polypeptide	−1,5	−2,8	−2,7	−2,1	−2,1	−2,4	−2,8
*Adcy6*	adenylate cyclase 6	−0,6	−0,7	−0,5	−0,7	−0,6	0	−0,1
*Sos1*	Son of sevenless homolog 1 (Drosophila)	−0,4	−0,4	−0,1	0	0	−0,1	0,2
*Mras*	muscle and microspikes RAS	0	−0,5	−0,6	−0,4	−0,2	0,7	0,5
*Vav3*	vav 3 oncogene	0	−0,1	−0,2	−0,1	−0,1	0,1	0
*Pak3*	p21 (CDKN1A)-activated kinase 3	0,3	−0,2	−0,7	−0,9	−0,5	−0,3	−0,9
*Wasf2*	WAS protein family, member 2	−0,2	−0,7	−0,8	−0,8	−0,3	0,1	0
*Limk2*	LIM motif-containing protein kinase 2	−0,1	0,1	0,1	0	0,1	0,7	1,4
*Arp2/3*	Actin related protein 2/3	−0,1	0,1	0,3	0,5	0,8	0,5	1

Fold changes are presented relative to 0 h (log_2_-transformed ratios).
