# Supplementary material for: Inhibition of fat cell differentiation in 3T3-L1 pre-adipocytes by all-trans retinoic acid: Integrative analysis of transcriptomic and phenotypic data
Source: Biomol Detect Quantif. 2016 Nov 21;11:31–44. doi: 10.1016/j.bdq.2016.11.001 (PMC5348118; doi:10.1016/j.bdq.2016.11.001)

**Supplement material = in total 7 figures and 8 tables**

**Supplement Figure 1: For data validation, the microRNA expression of three putative adipogenesis relevant microRNAs in DMI-treated (red) and DMI + 3 µM ATRA-treated (blue) preadipocytes was analysed and verified by RT-qPCR experiments. (A)** *miR-103* **(B)** *miR-146* and **(C)** *miR-221***.** Expression changes in ATRA-untreated 3T3-L1 cells are presented relative to 0 h, whereas the expression changes in ATRA-treated 3T3-L1 cells are presented relative to the corresponding untreated samples (log_2_-transformed ratios).


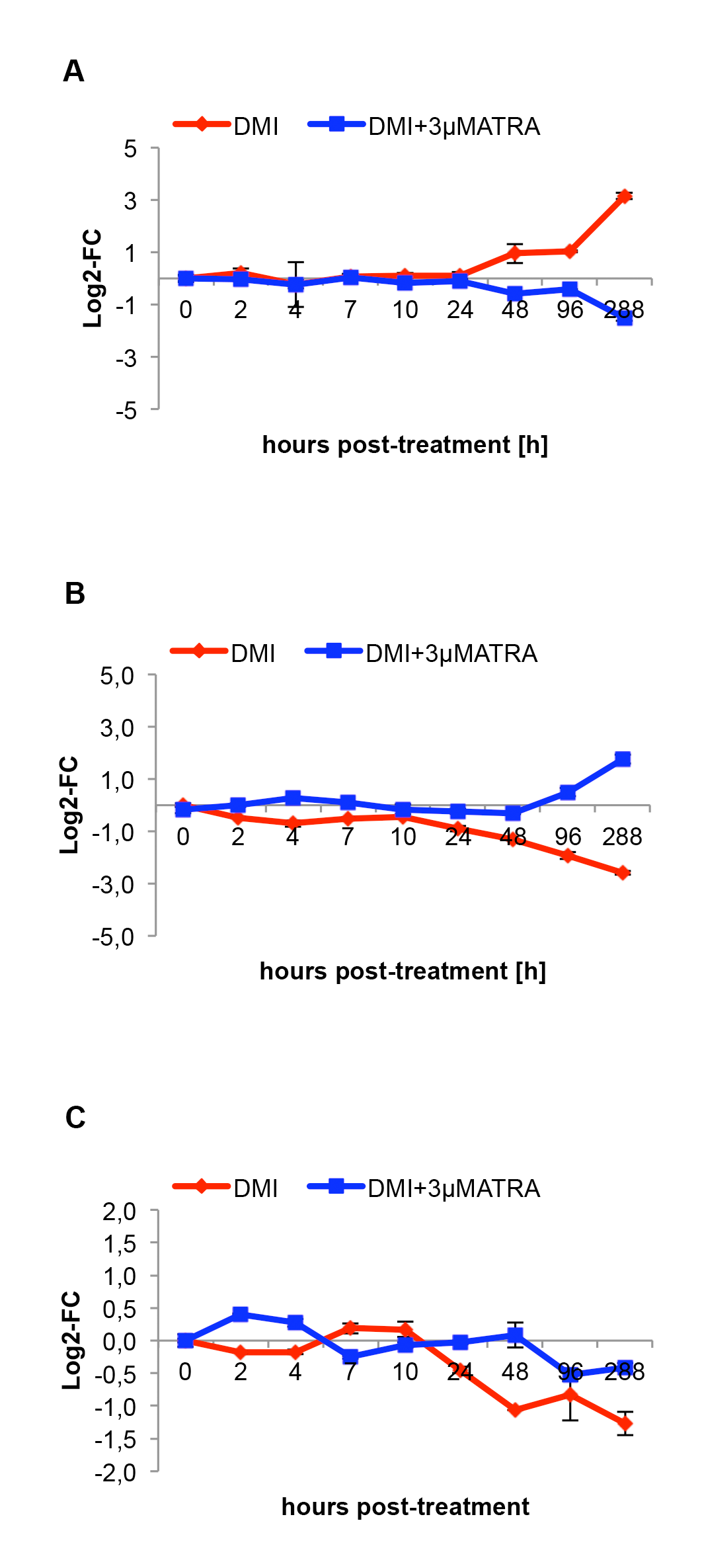


**Supplement Figure 2: For data validation, the microRNA expression of five highly regulated microRNAs in DMI-treated (red) and DMI + 3 µM ATRA-treated (blue) preadipocytes was analysed and verified by RT-qPCR experiments. (A)** *miR-29a* **(B)** *miR-29b* **(C)** *miR-365* **(D)** *miR-93* and **(E)** miR-96**.** Expression changes in ATRA-untreated 3T3-L1 cells are presented relative to 0 h, whereas the expression changes in ATRA-treated 3T3-L1 cells are presented relative to the corresponding untreated samples (log_2_-transformed ratios).


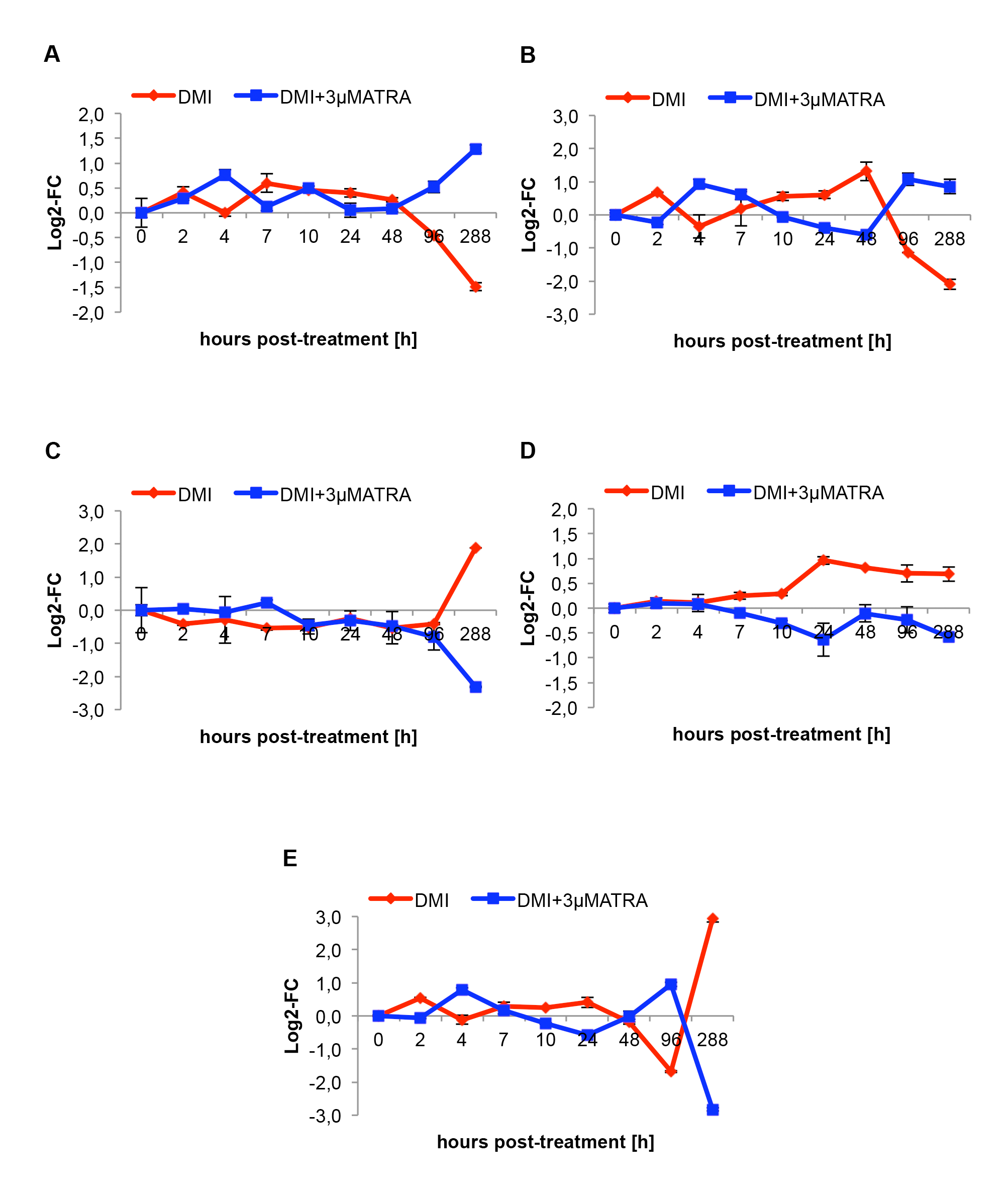


**Supplement Figure 3: Linear regression. (A)** Correlation of log_2_ FC microRNA gene expression levels by Nanostring analysis versus confirmatory RT-qPCR (r = 0.8). **(B)** Correlation of fold-change mRNA gene expression levels (log_2_ FC) by Affymetrix microarray analysis versus confirmatory RT-qPCR (r = 0.7).


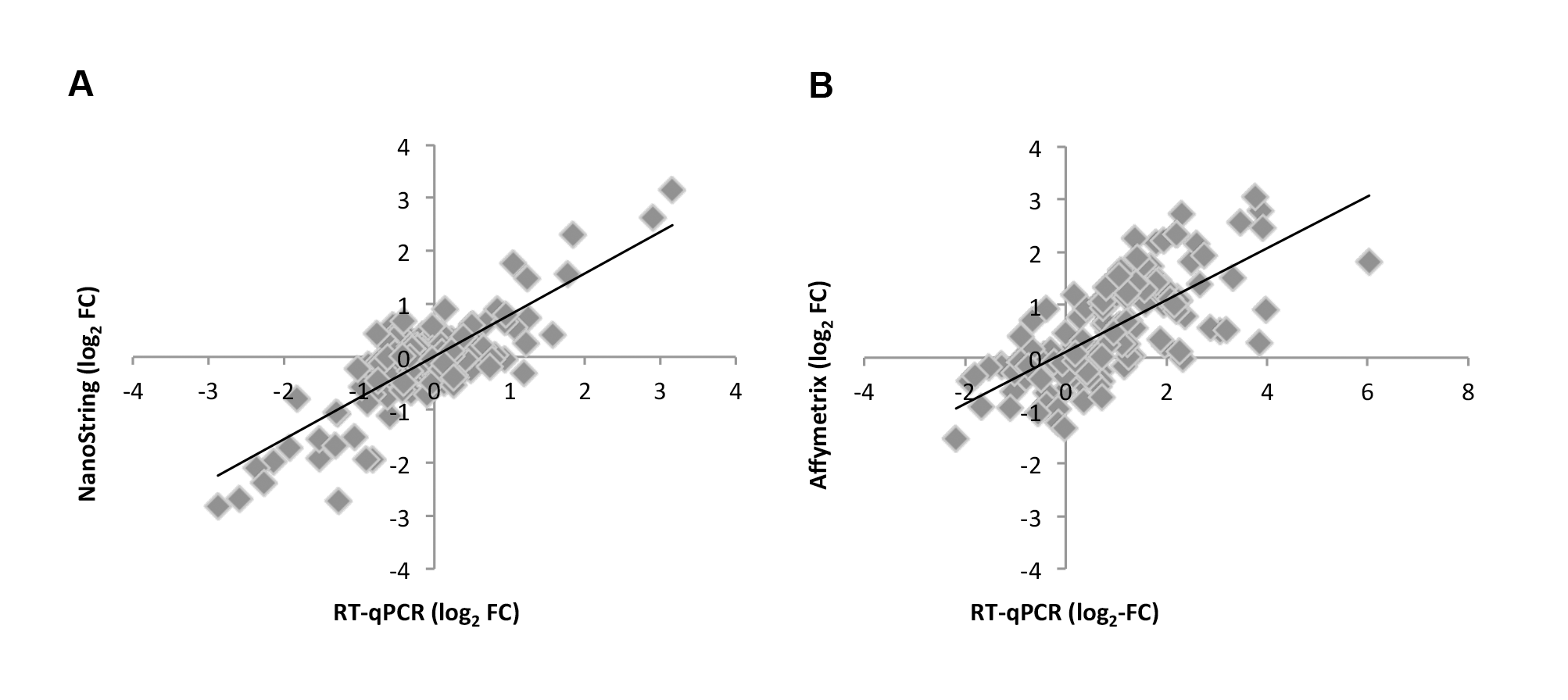


**Supplement Figure 4: GO clustering.** GO terms from 0 h to 96 h post-induction with ATRA were analysed by the Genomatix Software, and the top 50 regulated GO terms per time were selected and visualised with Revigo (42). **(A)** GO clustering after 2, 4 and 7 h post-induction with DMI + 3 µM ATRA (early regulation). **(B)** GO clustering after 10 and 24 h post-induction with DMI + 3 µM ATRA (mid regulation). Each rectangle presents the log_10_ *p*-value of a GO term. The log_10_ *p*-value for each GO is also represented.


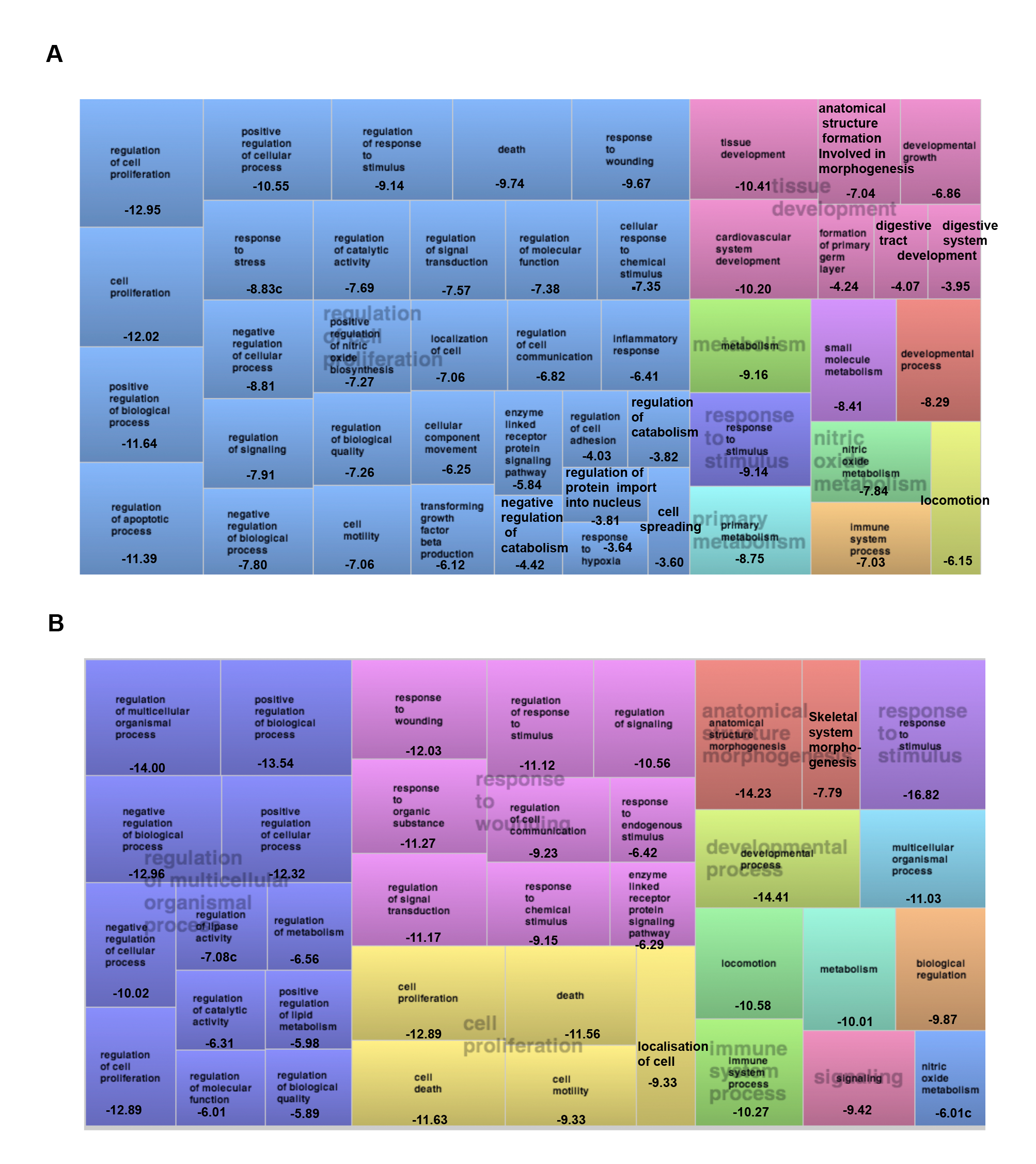


**Supplement Figure 5: Joint analysis of real microRNA and mRNA data on the basis of the target prediction software TargetScan Mouse 5.2** (44) **and a linear regression model** (49)**.** The edges in the network correspond to an association of miRNA to the genes at the sequence and expression level. The edge weights denote the negative coefficient of the respective miRNA–mRNA relation in the multiple regression model. The functional locality score of 2.86E-06 is a measure of the local over-representation within the network of **gap junction signalling**. Nodes with a diamond shape are directly associated with the respective process. The colour of the nodes corresponds to the *p*-value for the test of over-representation of the process in the surrounding of the node, i.e. all nodes that are targeted by the same miRNA.


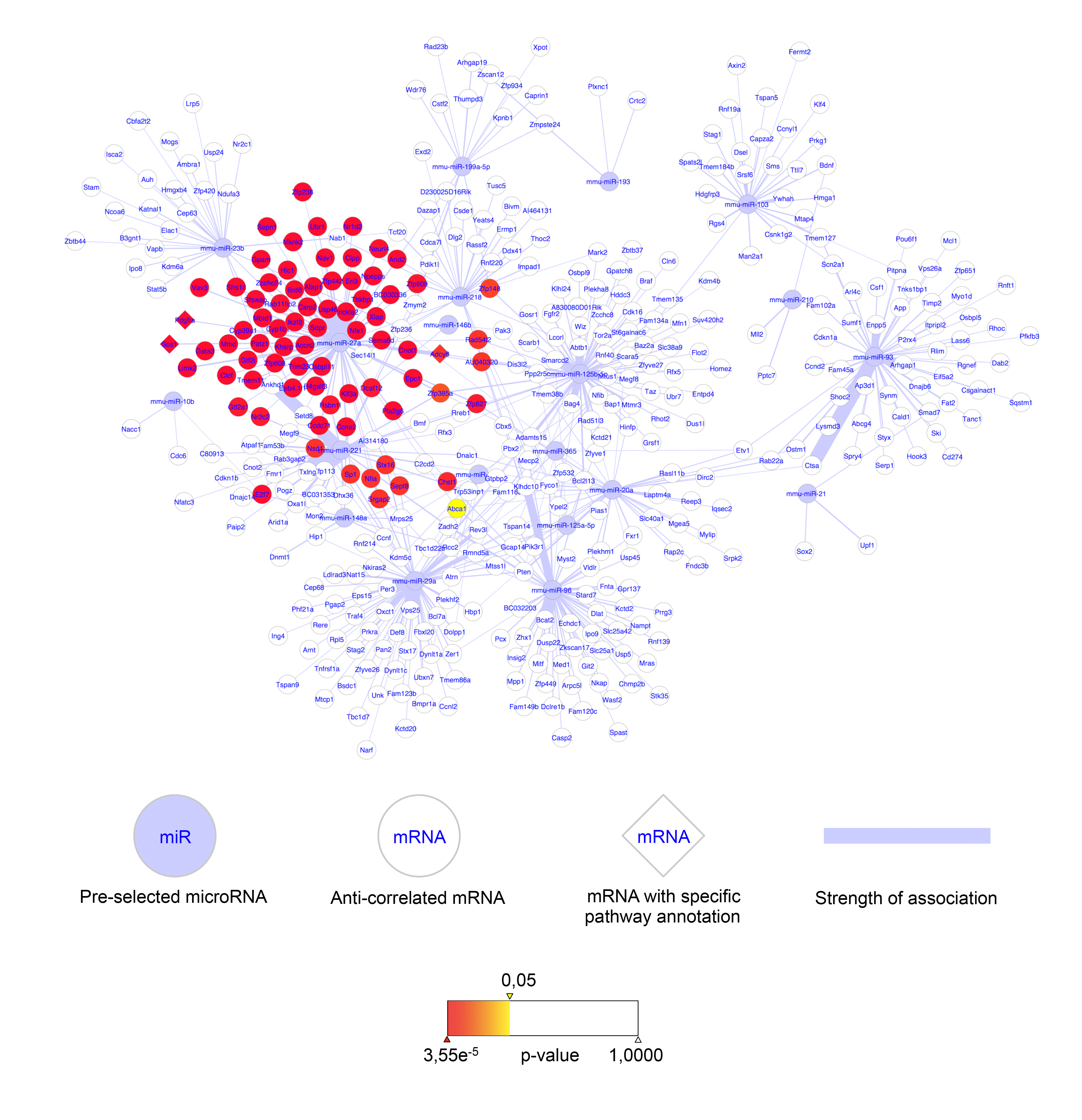


**Supplement Figure 6: Joint analysis of real microRNA and mRNA data on the basis of the target prediction software TargetScan Mouse 5.2** (44) **and a linear regression model** (49)**.** The edges in the network correspond to an association of miRNA to the genes at the sequence and expression level. The edge weights denote the negative coefficient of the respective miRNA–mRNA relation in the multiple regression model. The functional locality score of 1.06E-10 is a measure of the local over-representation within the network for **the rearrangement of the actin cytoskeleton.** Nodes with a diamond shape are directly associated with the respective process. The colour of the nodes corresponds to the *p*-value for the test of over-representation of the process in the surrounding of the node, i.e. all nodes that are targeted by the same miRNA.


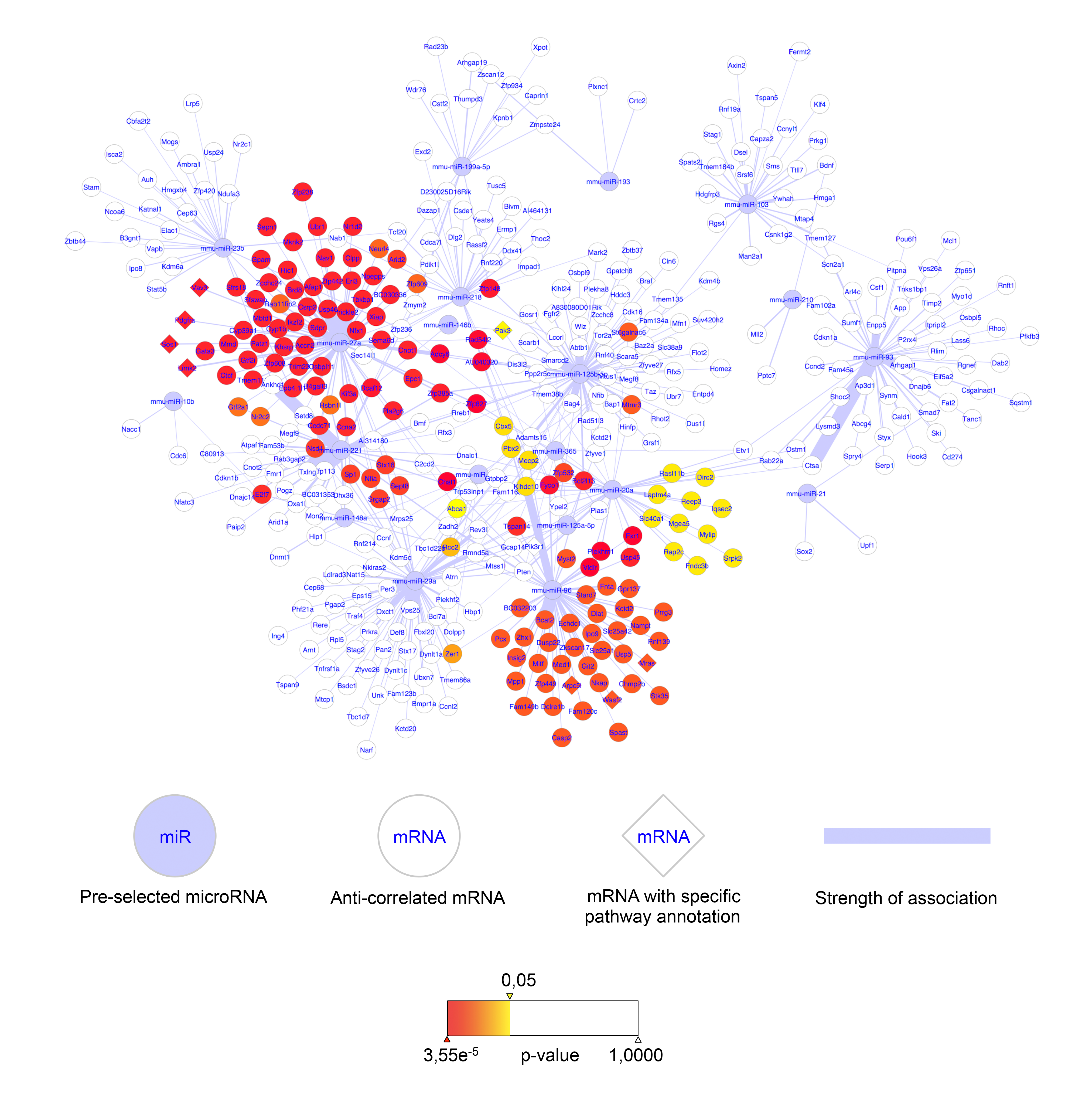


**Supplement Figure 7: Joint analysis of real microRNA and mRNA data on the basis of the target prediction software TargetScan Mouse 5.2** (44) **and a linear regression model** (49)**.** The edges in the network correspond to an association of miRNA to the genes at the sequence and expression level. The edge weights denote the negative coefficient of the respective miRNA–mRNA relation in the multiple regression model. The functional locality score of 5.84E-26 is a measure of the local over-representation within the network of the TCA cycle. Nodes with a diamond shape are directly associated with the respective process. The colour of the nodes corresponds to the *p*-value for the test of over-representation of the process in the surrounding of the node, i.e. all nodes that are targeted by the same miRNA.


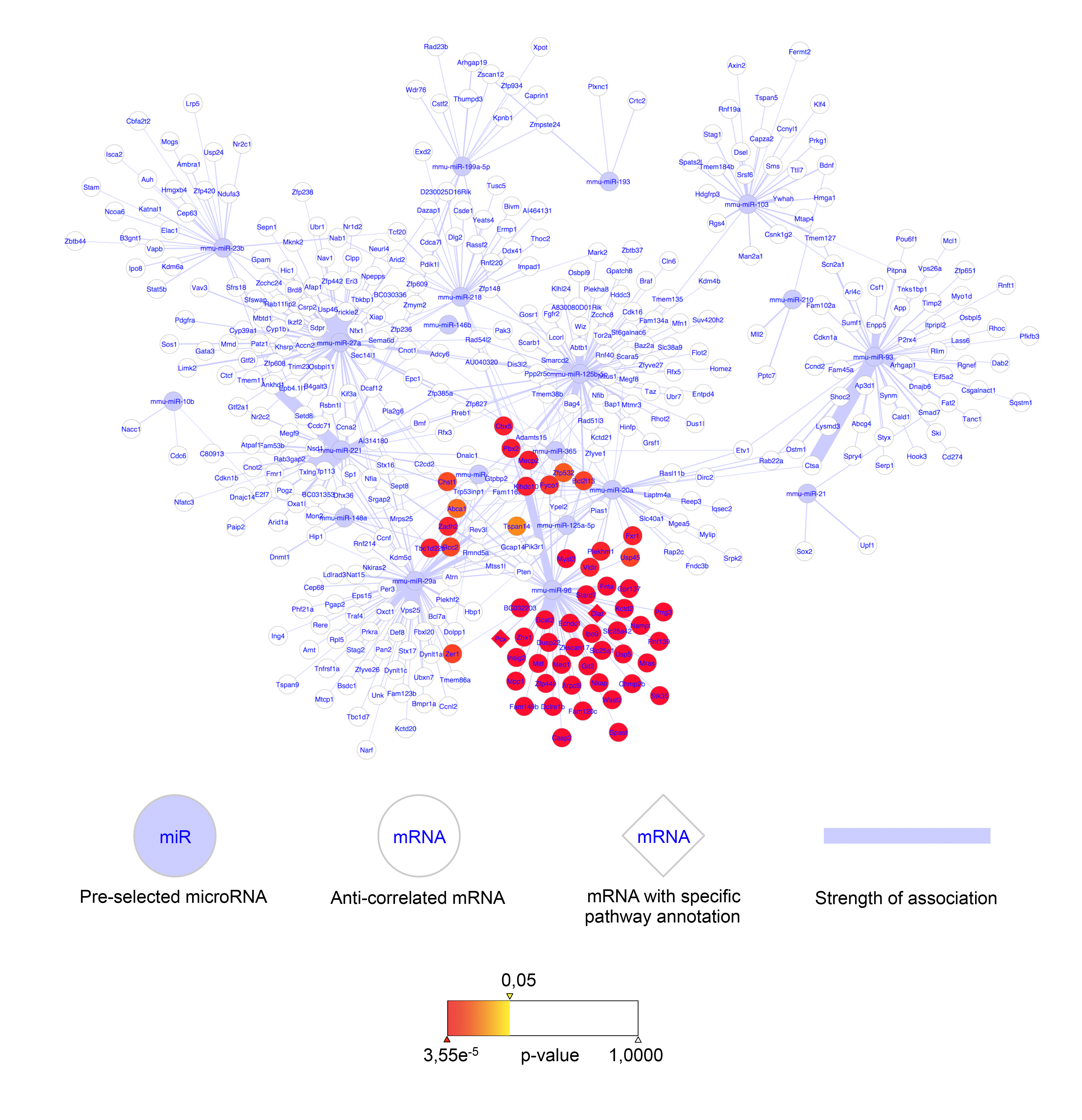


**Supplement Tables:**

**Supplement Table 1: List of gene name, forward and reverse primer sequences and product size used for RT-qPCR.**

**
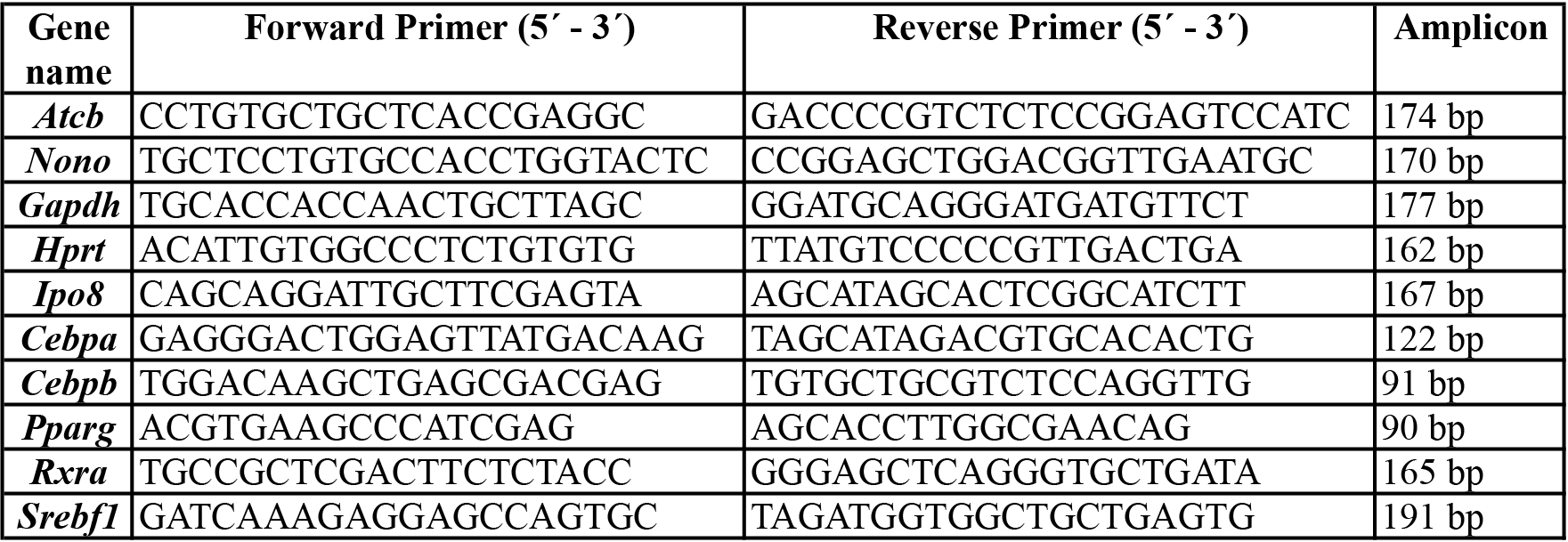
**

**Supplement Table 2: Name and sequence of measured mature microRNAs.**

**
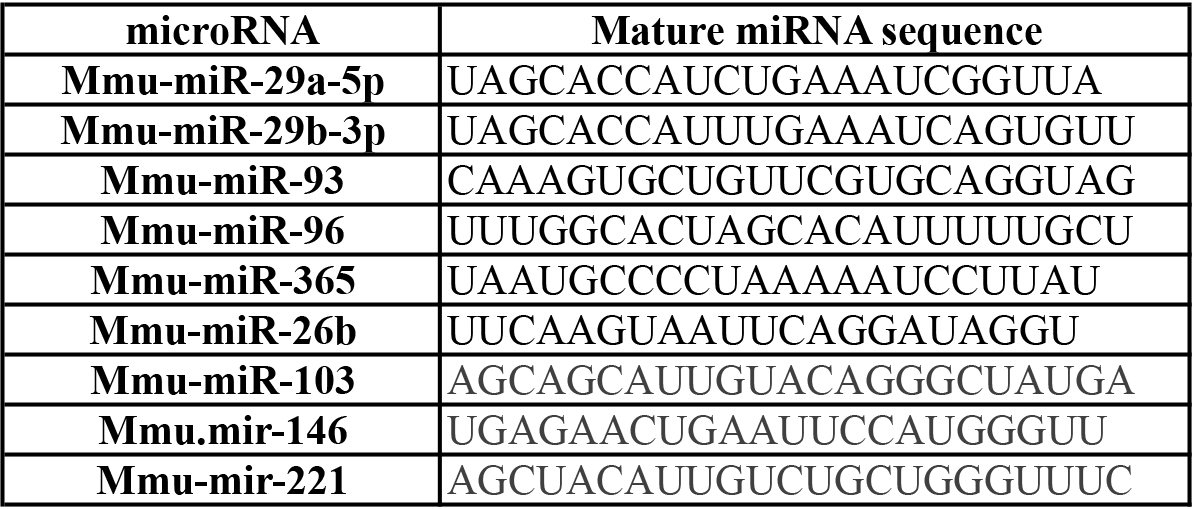
**

**Supplement Table 3: RT-qPCR results for microRNA expression data validation in DMI-treated 3T3-L1 cells.** Fold changes are presented relative to 0 h (log_2_-transformed ratios).


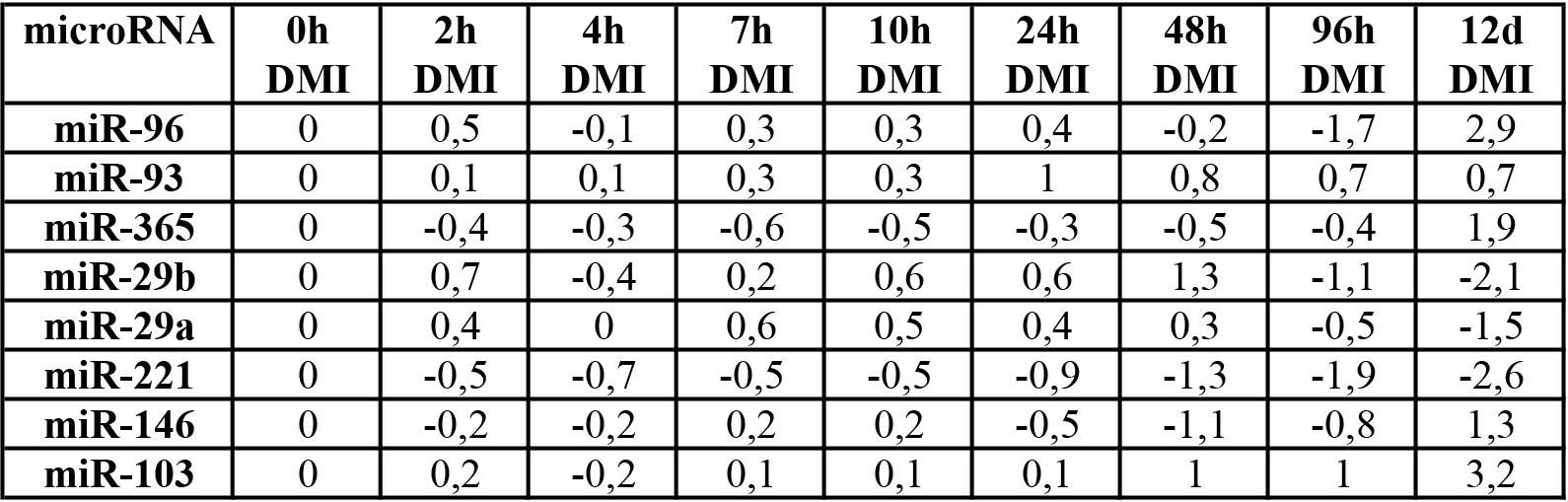


**Supplement Table 4: RT-qPCR results for microRNA expression data validation in ATRA-treated 3T3-L1 cells.** Fold changes are presented relative to the corresponding untreated samples (log_2_-transformed ratio).


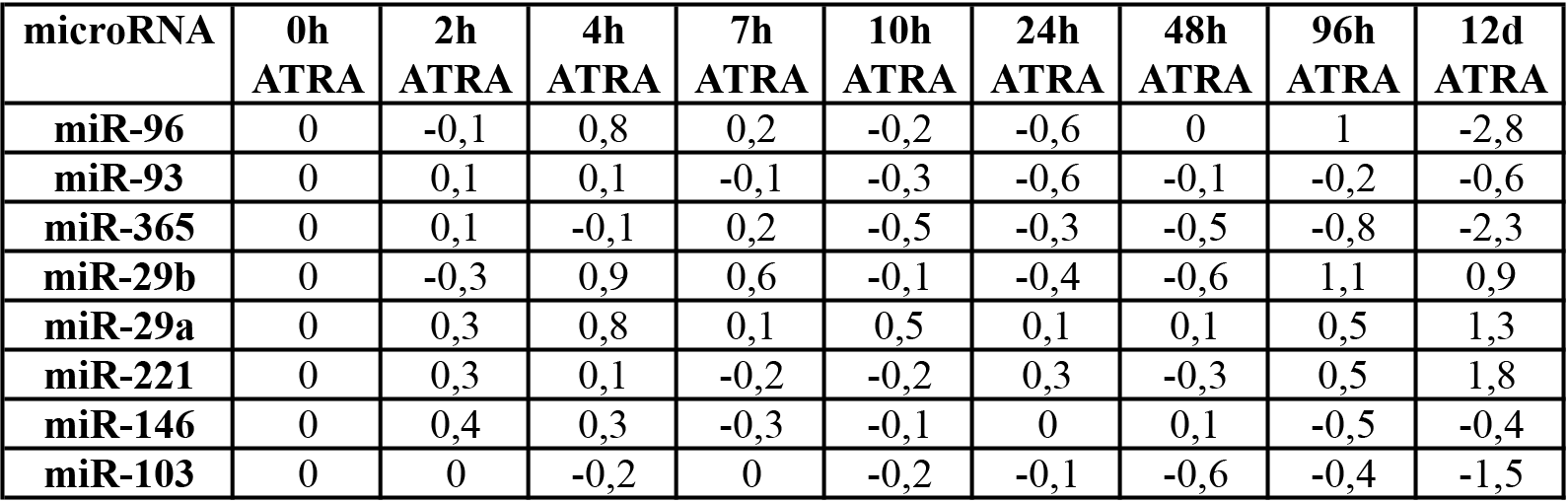


**Supplement Table 5: Expression values of five pre-selected genes out of the Affymetrix microarray experiments for DMI-treated 3T3-L1 cells.** Fold changes are presented relative to 0 h (log_2_-transformed ratios).


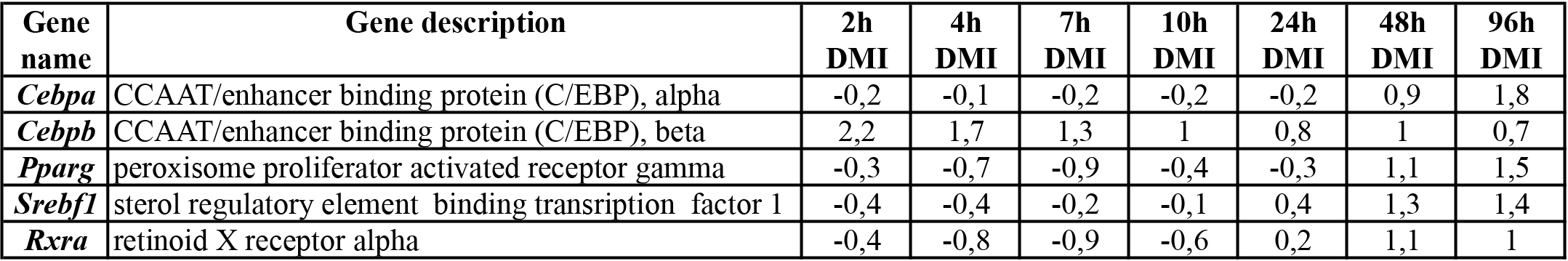


**Supplement Table 6: Expression values of five selected genes out of the Affymetrix microarray experiments for DMI + 3 µM ATRA-treated 3T3-L1 cells.** Fold changes are presented relative to the corresponding untreated samples (log_2_-transformed ratio).


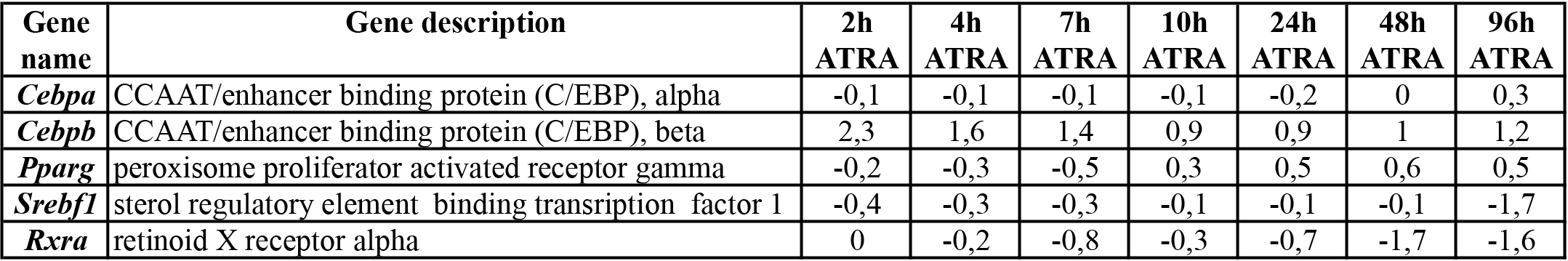


**Supplement Table 7: RT-qPCR results for the validation of the mRNA expression data of the Affymetrix microarray experiments in DMI-treated 3T3-L1 cells.** Fold changes are presented relative to 0 h (log_2_-transformed ratios).

**
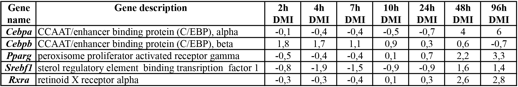
**

**Supplement Table 8: RT-qPCR results for the validation of the mRNA expression data of the Affymetrix microarray experiments in DMI + 3 µM ATRA-treated 3T3-L1 cells.** Fold changes are presented relative to the corresponding untreated samples (log_2_-transformed ratios).


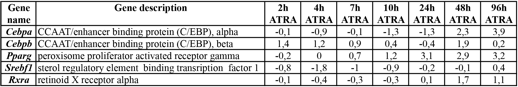

Supplement: Supplementary file 1 [file mmc1.docx]
